# Serological evidence of SARS-CoV-2 exposure in marine mammals in the United States between 2020 and 2025

**DOI:** 10.1371/journal.pone.0351734

**Published:** 2026-07-09

**Authors:** Idrissa Nonmon Sanogo, Wendy B. Puryear, Alexa F. Simulynas, Robert DiGiovanni, Caroline E. Goertz, Natalie Hunter, Kathy Burek Huntington, Natalie Rouse, Rosemary E. Seton, Nicole E. Hunter, Kim Schulam, Stephen J. St. Pierre, Lynda Doughty, Jay Pagel, Allison D. Tuttle, Sarah Callan, Marina A. Piscitelli-Doshkov, Lisa Becker, Brian Fadely, Sarah M. Chinn, Anthony J. Orr, Heather Ziel, Josh London, Jessica L. Huggins, Deborah Fauquier, Sarah Wilkin, Ainsley Smith, Margot Madden, Maxine A. Montello, Alissa C. Deming, Michelle R. Rivard, Ashley Stokes, Lauren McDowell, Joanna Daniel, Dyanna Lambourn, Suzanne Thurman, Michelle Barbieri, Claudia Cedillo, Jonathan A. Runstadler

**Affiliations:** 1 Department of Infectious Disease and Global Health, Cummings School of Veterinary Medicine, Tufts University, North Grafton, Massachusetts, United States of America; 2 Atlantic Marine Conservation Society, Hampton Bays, New York, United States of America; 3 Alaska SeaLife Center, Seward, Alaska, United States of America; 4 Alaska Veterinary Pathology Services, Eagle River, Alaska, United States of America; 5 Allied Whale, College of the Atlantic, Bar Harbor, Maine, United States of America; 6 International Fund for Animal Welfare, Yarmouth Port, Massachusetts, United States of America; 7 Marine Mammal Alliance Nantucket, Nantucket, Massachusetts, United States of America; 8 Marine Mammals of Maine, Brunswick, Maine, United States of America; 9 Marine Mammal Stranding Center, Brigantine, New Jersey, United States of America; 10 Mystic Aquarium, Mystic, Connecticut, United States of America; 11 North Carolina Aquariums Jennette’s Pier, Nags Head, North Carolina, United States of America; 12 National Marine Life Center, Buzzards Bay, Massachusetts, United States of America; 13 Marine Mammal Laboratory, Alaska Fisheries Science Center, National Marine Fisheries Service, NOAA, Seattle, Washington, United States of America; 14 Cascadia Research Collective, Olympia, Washington, United States of America; 15 National Oceanic and Atmospheric Administration, National Marine Fisheries Service, Office of Protected Resources, Silver Spring, Maryland, United States of America; 16 National Oceanic and Atmospheric Administration Fisheries, Gloucester, Massachusetts, United States of America; 17 National Aquarium, Baltimore, Maryland, United States of America; 18 New York Marine Rescue Center, Riverhead, New York, United States of America; 19 Pacific Marine Mammal Center, Laguna Beach, California, United States of America; 20 SR3 (SeaLife Response, Rehabilitation, and Research), Des Moines, Washington, United States of America; 21 Seacoast Science Center, Rye, New Hampshire, United States of America; 22 Virginia Aquarium and Marine Science Center Stranding Response Program, Virginia Beach, Virginia, United States of America; 23 Washington Department of Fish and Wildlife, Wildlife Program, Science Division, Olympia, Washington, United States of America; 24 Marine Education, Research and Rehabilitation Institute, Lewes, Delaware, United States of America; 25 Pacific Islands Fisheries Science Center, National Marine Fisheries Service, NOAA, Honolulu, Hawaii, United States of America; Central Laboratory for Evaluation of Veterinary Biologics, Agricultural Research Center, EGYPT

## Abstract

Natural infections of severe acute respiratory syndrome coronavirus 2 (SARS-CoV-2) have been documented in over 60 animal species, some distantly related. Several marine mammals have been predicted as highly susceptible to SARS-CoV-2 infection based on the homology of their ACE2 receptors to those of humans. To assess potential exposure of marine mammals to SARS-CoV-2, we conducted an opportunistic survey from 2020 to 2025 and tested 1,808 swabs and 378 serum samples from 21 marine mammal species in the United States. All the swabs tested by RT-qPCR were negative, indicating the absence of active infection. A low level of SARS-CoV-2 neutralizing antibodies was detected in three pinniped species, including *Phoca vitulina* (harbor seal, 13.6%, 95% CI: 5.2–27.4%), *Zalophus californianus* (California sea lion, 7%, 95% CI: 1.9–17.0%), and *Halichoerus grypus* (grey seal, 3.7%, 95% CI: 0.5–12.7%). These findings represent the first serological evidence of SARS-CoV-2 exposure in marine mammals in the United States, highlighting the need for continued monitoring of these populations and further research on SARS-CoV-2 transmission dynamics in wildlife.

## Introduction

Severe Acute Respiratory Syndrome Coronavirus 2 (SARS-CoV-2), the virus responsible for the COVID-19 pandemic, is known for its remarkable ability to infect a wide range of distantly related animal species. Natural infections of SARS-CoV-2 have been documented in over 60 animal species, including many free-ranging wildlife species [1,2]. Given the high transmissibility of SARS-CoV-2 and its endemic prevalence in humans, there is increasing concern regarding its potential spillover to new animal species, including marine mammals [3,4].

Several studies have confirmed the presence of SARS-CoV-2 genome fragments in domestic untreated wastewater and marine environments [5–8]. Poor wastewater management or natural disasters may lead to the discharge of untreated wastewater, potentially carrying infectious materials into natural water systems. Consequently, marine mammal species inhabiting coastal areas with contaminated natural water may be exposed to a variety of new pathogens [4,9]. However, to date, infectious SARS-CoV-2 has not been found in untreated or treated wastewater or aquatic environments [10,11]. Marine mammals can also be exposed to human viruses in various other ways, including activities involving direct contact with humans, such as during rehabilitation and field research, or in stochastic ways associated with fishing or recreational activities. The rehabilitation of stranded marine mammals requires close interaction between humans and animals, which could expose marine mammals to human viruses and potentially increase the risk of reverse zoonosis [12]. Rehabilitated animals could introduce a novel pathogen acquired during rehabilitation into marine environments, leading to potential consequences for naïve populations [13].

Numerous marine mammal species, including most pinniped and cetacean species, particularly *P. vitulina*, was predicted to be highly susceptible to SARS-CoV-2 infection based on in silico modeling of SARS-CoV-2 spike protein binding affinity to their ACE2 receptors [4,14,15]. To date, natural SARS-CoV-2 infection in marine mammal species has not been reported, and the potential effect of SARS-CoV-2 spillover to marine mammals remains unknown. However, only a limited number of coronaviruses have been described in marine mammals, primarily alphacoronaviruses and gammacoronaviruses identified in seals and cetaceans, including one associated with epizootic pneumonia and mortality in Pacific harbor seals (*Phoca vitulina richardii*) [16,17]. Importantly, no betacoronaviruses closely related to SARS-CoV-2 have been reported in these hosts.

Exposure of marine mammals to SARS-CoV-2 could have serious consequences, particularly for highly social species such as seals and sea lions, which interact in large groups [18]. Such exposure could facilitate their role as a new reservoir and pose a future zoonotic threat. Consequently, investigating the epidemiology of SARS-CoV-2 in marine mammal hosts and assessing potential spillover events among these species will provide valuable information for public health. Several studies have investigated the epidemiology of SARS-CoV-2 in terrestrial mammals [2,19–21], but research on its circulation in marine mammals remains limited. In this study, we conducted an opportunistic survey of SARS-CoV-2 at the human–animal interface in diverse marine mammal species, including animals admitted to rehabilitation facilities and those sampled during active capture-release studies along the U.S. east and west coasts and Alaska, to assess potential exposure in these populations.

## Methods

### Ethics statement

This study was carried out in strict accordance with the recommendations in the Guide for the Care and Use of Laboratory Animals of the National Institutes of Health. The study was approved by Tufts University Institutional Animal Care and Use Committee (Number: G2023-02). Active capture-release studies on the West Coast were conducted under NMFS Permit #22678. Active capture-release studies of Steller sea lions (*Eumetopias jubatus*) were conducted under NMFS Permit #22289 and NMFS Alaska IACUC approval NWAK 18−03, and work in the Alaska Maritime National Refuge under permit #74500-15-005. Active capture-release studies of *H. grypus* were conducted under NMFS Permit #21719 and #26939, and U.S. Fish and Wildlife Service, Eastern Massachusetts NWR Complex Special Use Permits #53514-FY2019-01 and #53510-FY2022-01. Sea otter samples were collected under USFWS LOA-PER0051451. Additional approvals included NOAA Permit 24359, SA-AKR-2020-04, SA-AKR-2023-02, and USFWS Permit MAPER0032559 (AVPS). Samples provided by the Alaska SeaLife Center were obtained under NOAA SA-AKR-2025-05 for non-ESA–listed species. NOAA MMPA/ESA Permit 24359 applied to all samples from ESA-listed species.

### Sample origin and collection

A total of 1,808 nasal, oral, and rectal swab samples from 21 marine mammal species and 378 serum samples from 6 pinniped, two cetacean and one mustelid species were collected between 2020 and 2025 (Table 1) through a network of collaborators across the U.S. Samples were collected opportunistically from both live and dead stranded animals under each responding organizations federal (with the National Oceanic and Atmospheric Administration’s National Marine Fisheries Service) and regional stranding agreement. A subset of species was also sampled as part of active capture-release studies for health or stock assessments under the Marine Mammal Protection Act (MMPA), permitting, and following established procedures for marine mammal sampling. No animals were anesthetized or euthanized for the purpose of this study. All animal handling was performed by trained marine mammal rehabilitation personnel following standard animal welfare protocols. As determined by each facility or researchers’ authorizations or permitting followed by feasibility of collection, an individual animal may have had multiple swabs collected and sera, only swab(s), or only sera collected (S1 Table). Individual swabs were immediately placed into vials containing viral transport medium (VTM) consisting of Medium 199 supplemented with bovine serum albumin, gentamicin, benzylpenicillin, nystatin, and sulfamethoxazole [22] and stored at −20 °C or below at each collecting facility until shipment to the laboratory. Whole blood samples were centrifuged, and serum was collected in cryovials and stored at −20°C.

**Table 1:** Samples tested for SARS-CoV-2 RNA and antibodies in various marine mammal species between 2020 and 2025 in the US.

	Species	Scientific name	# individual Animals		# swab samples		# serum samples	
			S†	A‡	S	A	S	A
Pinniped	California sea lion	*Zalophus californianus*	4	58	6	30		57
	Steller sea lion	*Eumetopias jubatus*	1	80	3	79		34
	Northern fur seal	*Callorhinus ursinus*	8	20	19			20
	Grey seal	*Halichoerus grypus*	213	203	503	258	54	154
	Harbor Seal	*Phoca vitulina*	252		559		44	
	Harp seal	*Pagophilus groenlandicus*	65		156		11	
	Hooded seal	*Cystophora cristata*	3		7			
	Northern elephant seal	*Mirounga angustirostris*	3	20	7	20		
	Pacific walrus	*Odobenus rosmarus divergens*	2		5			
	Ribbon seal	*Histriophoca fasciata*		11		11		
	Spotted seal	*Phoca largha*		14		14		
	Hawaiian monk seal	*Neomonachus schauinslandi*	8		12			
Cetacean	Harbor porpoise	*Phocoena phocoena*	7		12			
	Beluga whale	*Delphinapterus leucas*	3		7			
	Striped dolphin	*Stenella coeruleoalba*	2		4		1	
	Common dolphin	*Delphinus delphis*	6		21		1	
	Spotted dolphin	*Stenella frontalis*	1		2			
	Atlantic white-sided dolphin	*Leucopleurus acutus*	2		8			
	Minke whale	*Balaenoptera acutorostrata*	1		4			
	Humpback whale	*Megaptera novaeangliae*	2		6			
Mustelid	Northern sea otter	*Enhydra lutris kenyoni*	28		56		2	
Total			611	406	1396	412	113	265

† Stranded animals.

‡ Active capture-release animals.

### RNA extraction and SARS-CoV-2 RT-PCR testing

RNA was extracted from swab samples using the Mag-Bind Viral DNA/RNA Extraction Kit (Omega Bio-Tek, Norcross, GA, USA) on a KingFisher Flex platform, following the manufacturer’s protocol. All samples were tested for SARS-CoV-2 viral RNA using a real-time RT-PCR assay targeting the open reading frame 1b non-structural protein 14 (ORF1b-nsp14) region (HKU-ORF1 primers: Forward 5′-TGGGGYTTTACRGGTAACCT-3′, Reverse 5′-AACRCGCTTAACAAAGCACTC-3′, Probe 5′-FAM-TAGTTGTGATGCWATCATGACTAG-TAMRA-3′) [23]. Samples with a cycle threshold (Ct) value  <  40 were considered putatively positive for SARS-CoV-2 RNA.

Each batch of samples included a positive control (genomic RNA from SARS-CoV-2 isolate USA-WA1/2020, BEI resources NR-52281) and a negative control (VTM). All samples were further tested for evidence of viable RNA by detection of endogenously expressed β-actin (Ct  <  40) as previously described [24]. Animals with a minimum of one swab sample with detectable β-actin were considered valid for further interpretation regarding the presence or absence of SARS-CoV-2 RNA detection.

### Detection of SARS‐CoV‐2 antibodies by ELISA-RBD

SARS-CoV-2 antibodies from serum were detected using an indirect pan-mammalian ELISA specifically targeting the SARS-CoV-2 receptor binding domain (RBD), following a previously described protocol [24]. The ELISA assay was initially validated in-house for detecting total immunoglobulins in serum samples across multiple mammalian orders, including marine mammals (S1 Fig).

Immulon 4 HBX plates (part # 3855) were coated with purified SARS-CoV-2 RBD protein (NR-52366, BEI Resources) at a concentration of 2 µg/ml and incubated at 4 °C for 24 hours. After incubation, plates were washed three times with phosphate-buffered saline containing 0.1% Tween-20 (PBS-T) and blocked with Pierce Protein-Free Blocking Buffer (Thermo Fisher, catalog no. PI37573) for 1 hour at room temperature. Serum samples were initially diluted 1:5 in PBS in separate plates, then transferred to the ELISA plates to achieve a final dilution of 1:50 in PBS-T supplemented with 1% milk. Plates were incubated for 2 hours at room temperature. Following incubation, plates were washed three times with PBS-T, and 50 μL of the secondary antibody, Pierce Recombinant Peroxidase-Conjugated Protein A/G (Thermo Fisher, catalog no. 32490), diluted 1:10,000 in PBS-T containing 1% milk, was added to each well. Plates were incubated for 1 hour at room temperature. Plates were then washed and developed with SigmaFast OPD tablets (Sigma-Aldrich catalog no. P9187).

Absorbance values were measured at 490 nm on a BioTek Synergy 4 Multidetection Plate Reader, with the positive cutoff defined as the mean plus three standard deviations (μ + 3σ) of the negative controls (n = 8). All samples were run in duplicate, and each plate included serum from spike (S) protein-immunized alpacas as a positive control and serum from uninfected *H. grypus* obtained before the COVID-19 pandemic in 2018 as a negative control (S2 Fig). Positive samples from the initial ELISA-RBD screening were validated by a confirmatory ELISA using 3-fold serial dilutions starting at 1:100 against the full-length SARS-CoV-2 spike protein (BEI Resources, NR-52308). Positives were defined as samples showing a signal greater than the mean plus three standard deviations (μ + 3σ) of the negative controls in at least the first two consecutive dilutions [25].

### SARS‐CoV‐2 neutralization assay

A virus neutralization test (VNT) to validate the ELISA-positive samples (n = 18) was performed under BSL-3 conditions at the New England Regional Biosafety Laboratory (NERBL). Heat-inactivated (56 °C, 30 min) serum samples were serially diluted two-fold, starting at 1:4, in duplicate. Sera dilutions were incubated with 200 TCID50 of SARS-CoV-2 isolate USA-WA1/2020 (BEI resources, NR-52281) for 1 h at room temperature. The mixtures were subsequently added to confluent Vero E6 cells (ATCC-CRL-1586) in 96-well plates and maintained at 37 °C with 5% CO₂. Each plate included a virus-only control, a cell-only control, and a serum-only control. Cytopathic effect (CPE) was independently examined under a light microscope by two operators at 5 days post-infection, and the neutralization titer was recorded as the highest serum dilution that provided 100% neutralization of the reference virus and completely prevented CPE in both duplicate wells. Neutralizing titers equal to or greater than 16 were considered seropositive. An animal was considered seropositive only if the corresponding sample tested positive by both ELISA and VNT.

**Statistical analyses**: SARS-CoV-2 seropositivity rate was calculated as the proportion of seropositive animals among the total number of individuals tested for each species. Exact 95% confidence intervals were estimated using the binomial distribution. Differences in seropositivity rates among species were assessed using Fisher’s exact test. All statistical analyses were performed in R (version 2025.05.1; R Foundation for Statistical Computing, Vienna, Austria), and statistical significance was defined as p < 0.05.

## Results

A total of 1,017 individual animals were tested. Among these, 639 were tested only for SARS-CoV-2 RNA, 163 were tested only for antibodies, and 215 were tested for both viral RNA and antibodies. Of the 1,808 total swabs tested, 1,207 swabs tested positive for β-actin by RT-qPCR. This represents at least one viable sample from 656 of the 854 individual animals tested, confirming the presence of amplifiable RNA in 76.8% of the overall animals tested (S2 Table). Detection of endogenous β-actin was successful in the majority of pinniped species tested (9/12), with *Z. californianus*, *E. jubatus*, and *M. angustirostris* as the exceptions. The detection of endogenous β-actin was also successful in the majority of cetacean species (7/9) and in *E. lutris kenyoni* (S2 Table). All swabs were negative for SARS-CoV-2 RNA (S1 Table), indicating the absence of detectable SARS-CoV-2 RNA in marine mammal samples collected from 2020 to 2025.

**Fig 1:**
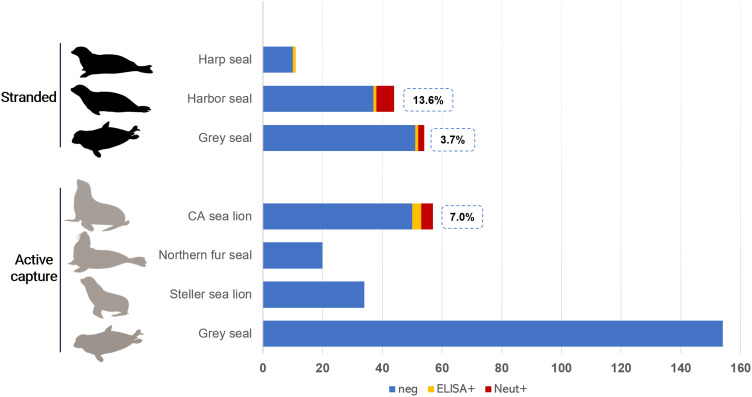
Detection of SARS-CoV-2 antibodies in pinnipeds.

In contrast, 18 out of 378 (4.8%; 95% CI: 2.6–6.9%) serum samples tested positive for binding antibodies against the SARS-CoV-2 RBD, including seven *P. vitulina*, three *H. grypus*, one *P. groenlandicus*, and seven *Z. californianus* (Fig 1). Of those, 12 also tested positive in a separate confirmatory ELISA against SARS-CoV-2 full spike protein, four tested negative, and two were untested due to limited sample volume. Neutralizing antibodies against SARS-CoV-2 WA1/2020 were detected in nine of the 12 ELISA spike-positive samples, as well as in three additional samples, two not tested by Spike ELISA and one Spike ELISA-negative sample, with neutralization titers ranging from 1:16–1:128 (Table 2).

**Table 2:** Seropositive marine mammal samples.

Common name	Animal ID	animal status	Sample date	State	RBD ELISA	Spike ELISA	Neut titer
Z. californianus	SMI23-21	active capture-release	3/20/2023	CA	P	NT	1:32
Z. californianus	SMI23-11	active capture-release	3/20/2023	CA	P	P	1:64
Z. californianus	SMI24-C8	active capture-release	3/21/2024	CA	P	N	1:32
Z. californianus	SMI24-C16	active capture-release	3/21/2024	CA	P	P	<1:8
Z. californianus	SMI24-C17	active capture-release	3/21/2024	CA	P	N	<1:8
Z. californianus	SMI24-C19	active capture-release	3/21/2024	CA	P	P	1:16
Z. californianus	2129	active capture-release	3/21/2024	CA	P	N	<1:8
P. vitulina	NA25012Pv	stranded	11/18/2021	DE	P	P	1:128
P. vitulina	SSC23-081Pv	stranded	9/23/2023	MA	P	P	1:32
P. vitulina	NMLC23-020Pv	stranded	6/30/2023	MA	P	P	<1:8
P. vitulina	SSC23-114Pv	stranded	11/13/2023	MA	P	P	1:16
P. vitulina	NMLC23-019Pv	stranded	6/27/2023	MA	P	NT	1:16
P. vitulina	IFAW23-195Pv	stranded	7/05/2023	MA	P	P	1:128
P. vitulina	SR323-36Pv	stranded	10/9/2023	WA	P	P	1:16
H. grypus	SSC23-039Hg	stranded	6/25/2023	NH	P	P	1:128
H. grypus	NMLC23-012Hg	stranded	5/20/2023	ME	P	P	<1:8
H. grypus	SXHG2356	stranded	6/28/2023	RI	P	P	1:16
P. groenlandicus	NA22017Pg	stranded	3/11/2022	MD	P	N	<1:8

**RBD ELISA (SARS-CoV-2 ELISA to RBD domain) and Spike ELISA (SARS-CoV-2 ELISA to spike protein): P** = positive; **N** = negative; **NT** = not tested. Neutralization titer is the serum dilution at which duplicate samples neutralized SARS-CoV-2 WA1/2\020. All positive detections are shaded in grey.

**Fig 2:**
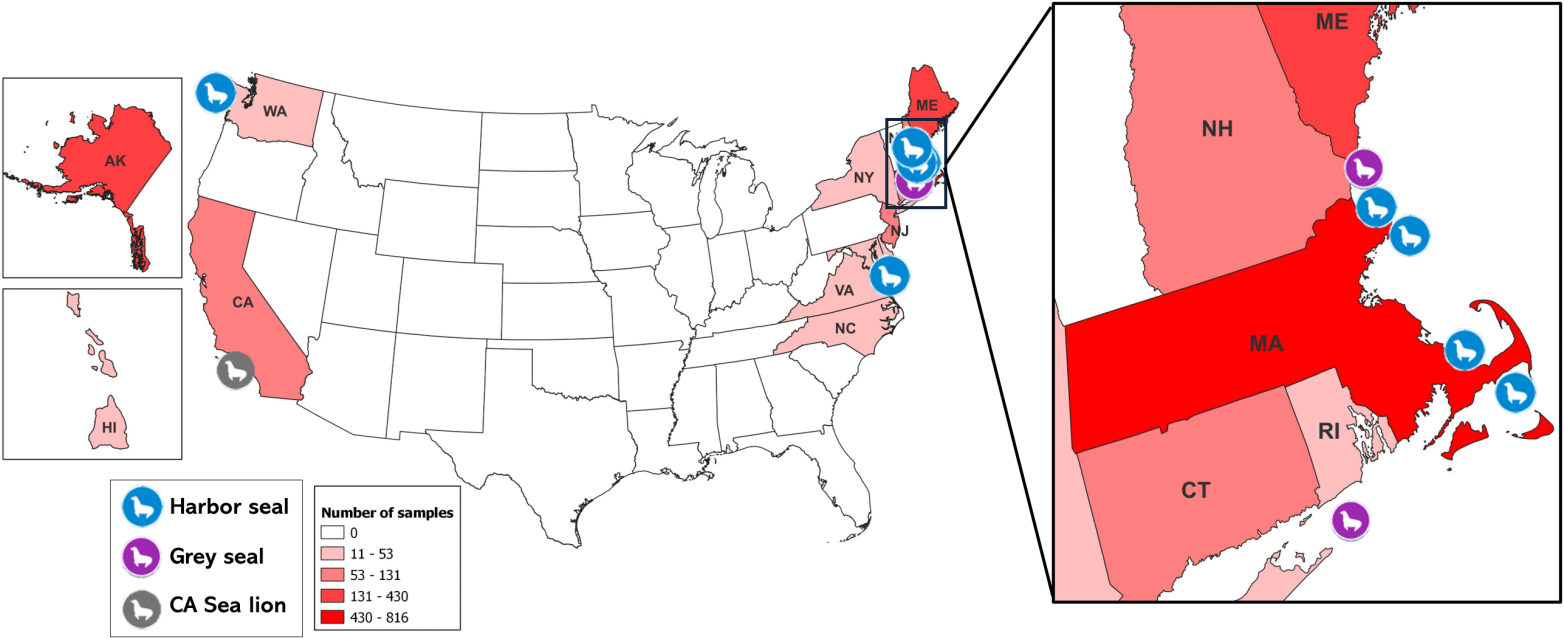
Map showing sample collection sites and location of pinnipeds with SARS-CoV-2 neutralizing antibody detections in the United States. Color intensity indicates the relative number of samples collected per state. SARS-CoV-2 neutralizing antibodies were detected in three marine mammal species, including *Phoca vitulina* (blue), *Halichoerus grypus* (purple), and *Zalophus californianus* (Gray). State boundaries were derived from public domain U.S. Census Bureau TIGER/Line shapefiles [26].

Seropositive samples were detected in *P. vitulina*, *H. grypus*, *P. groenlandicus*, and Z. californianus. In *H. grypus*, no neutralizing antibodies were detected in samples collected during capture-release studies. In all three seal species, one of the seropositive samples failed to neutralize SARS-CoV-2 WA1/2020, leaving 6/7 *P. vitulina*, 2/3 *H. grypus*, and 0/1 *P. groenlandicus* with sera that both bound to SARS-CoV-2 antigens on ELISA, and also neutralized whole live virus (Table 2). Nearly all of these detections were from the northeastern United States in 2023, with a single neutralizing antibody-positive *P. vitulina* detected in the Pacific Northwest in October 2023 (Fig 2). The earliest detection of a positive neutralizing antibody sample occurred in a single *P. vitulina* in the mid-Atlantic in November 2021. *P. vitulina* SSC23–081 had sera collected on 2 occasions, 8 days apart. Both samples from that animal were seropositive with comparable values on all serology assays.

For Z. californianus, seropositive samples were detected against the SARS-CoV-2 RBD antigen in 7 animals that were part of active capture-release efforts on San Miguel Island, CA, during the springs of 2023 and 2024. 3/6 seropositive samples also bound to SARS-CoV-2 spike protein in a separate analysis, and 4/7 neutralized whole SARS-CoV-2 WA1/2020 (Table 2).

Overall, the highest percentage of animals with neutralizing antibodies against SARS-CoV-2 WA1/2020 was observed in stranded *P. vitulina* (13.6%, 95% CI: 5.2–27.4%, N  =  44), followed by actively captured and released *Z. californianus* (7%, 95% CI: 1.9–17.0%, N  = 57), and stranded *H. grypus* (3.7%, 95% CI: 0.5–12.7%, N  = 54). No statistically significant differences in seropositivity were detected among the three species.

No SARS-CoV-2 neutralizing antibodies were detected in serum samples collected from active capture-release studies for *E. jubatus* (n = 34), or *C. ursinus* (n = 20), nor during sampling of stranded *P. groenlandicus* (n = 11), *D. delphis* (n = 2), and *E. lutris kenyoni* (n = 2). The overall percentage of SARS-CoV-2 neutralizing antibodies in all pinniped samples tested across species, geographies, and years was 3.2% (95% CI: 1.7–5.5% N = 378).

Species are clustered by stranded sampling (black silhouettes) and active capture-release sampling (grey silhouettes). Bars show the total number of sera samples tested, with one sample per animal. Seronegative samples are shown in blue, SARS-CoV-2 RBD ELISA positive samples are shown in orange, and SARS-CoV-2 WA1/2020 neutralizing antibody positive samples are shown in red. The percentage of neutralizing antibody positives is shown in the box.

## Discussion

To date, this study provides the first investigation of SARS-CoV-2 in several marine mammal species on both the East and West coasts of the U.S. We tested samples from a total of 1,018 individual animals representing 21 marine mammal species, sampled from live and dead stranded animals and during active capture-release studies. We found no evidence of SARS-CoV-2 viral RNA in any of the swab samples tested, indicating the absence of active infection in these species at the time of sampling. Consistent with our findings, SARS-CoV-2 surveillance studies of cetaceans stranded along the Italian coast (2020–2021) and pinnipeds stranded in Brazil also reported no evidence of viral RNA detection [14,27]. Interestingly, although no active infection was detected in any of the marine mammals tested, we found neutralizing antibodies to SARS-CoV-2 at a low seroprevalence (3.2% overall) in three marine mammal species, including the *Z. californianus*, *H. grypus*, and *P. vitulina*, sampled from different locations, indicating their exposure to the virus.

It remains unclear how these animals came into contact with the virus. However, SARS-CoV-2 transmission from humans to wildlife, particularly white-tailed deer, has been reported in multiple instances, highlighting the capacity of the virus for reverse zoonosis [2,28,29]. Cross-species transmission of pathogens from humans to marine mammals has been documented [30,31]. For example, the human influenza A(H1N1)pdm09 virus was detected in free-ranging northern elephant seals, supporting the possibility that human respiratory viruses can spill over into marine mammals [31,32]. It has been suggested that these seals may have been exposed to the H1N1 virus through feces discharged from sewage-dumping ships. A similar mechanism could be hypothesized for SARS-CoV-2 exposure in marine mammals, as the virus is shed in the feces of infected humans [33] and cruise ship traffic has increased in recent years in the areas we sampled. Nevertheless, the rapid loss of SARS-CoV-2 infectivity in feces suggests that contaminated human waste is unlikely to represent a major transmission route [34]. In contrast, direct contact between humans and marine mammals could provide opportunities for cross-species transmission. The samples tested in this study were primarily collected from animals in rehabilitation facilities, where close human-animal interactions are common [13]. One of the seropositive *P. vitulina* in this study, with a high neutralizing antibody titer (animal ID - NA25012Pv), was a juvenile male admitted to a rehabilitation facility after stranding in Delaware with severe injuries and ingested fishing gear requiring surgical intervention. Although the animal ultimately died following complications from surgery, its rehabilitation history involved prolonged care and close contact with animal care personnel, which may have provided opportunities for human-to-animal exposure to SARS-CoV-2. Therefore, we cannot rule out the possibility that seropositive animals were exposed to infected humans during rehabilitation, as serum samples were not routinely collected before admission to the rehabilitation centers, and asymptomatic human individuals may have gone undetected.

In this study, we found the highest SARS-CoV-2 seropositivity rate (13.6%) in *P. vitulina*. Notably, *P. vitulina* was predicted to be highly susceptible to SARS-CoV-2 compared to other pinnipeds based on the homology of their ACE2 viral receptors with human ACE2 receptors, suggesting their potential risk of infection by SARS-CoV-2 if exposed to the virus [4]. Moreover, an epizootic pneumonia outbreak and the associated die-off of *P. vitulina* were linked to infection with another coronavirus (harbor seal coronavirus – HSCoV) detected in archived tissues during a retrospective study [17]. However, the clinical significance of the HSCoV remains unclear due to incomplete sampling during the outbreak. Nevertheless, this finding demonstrates that coronaviruses can infect marine mammals and may be associated with disease outbreaks.

Unexpectedly, SARS-CoV-2 neutralizing antibodies were detected in *Z. californianus* sampled during active capture-release studies, albeit at low titers, despite this species being predicted to have very low susceptibility to infection based on ACE2 binding models [4]. However, while in silico predictions provide useful insight into receptor compatibility, SARS‑CoV‑2 has demonstrated its remarkable ability to infect a broad range of mammalian hosts, indicating that factors beyond ACE2 binding affinity contribute to host susceptibility [35]. Moreover, the *Z. californianus* sampled in this study were captured and released in San Miguel Island, where they may interact with humans through activities such as ecotourism and research operations. These human-animal interfaces may increase opportunities for exposure to SARS-CoV-2 through spillover from infected individuals [36,37].

Although SARS-CoV-2 neutralizing antibodies have been detected in stranded *H. grypus*, no evidence of exposure has been observed in actively captured and released individuals. Notably, *H. grypus* sampled during active capture-release studies are typically healthy, newly weaned pups, which may have limited opportunities for exposure to the virus.

The majority of seropositive animals were identified on the East Coast; however, this pattern likely reflects the geographic distribution and nature of our sampling rather than a true regional difference in exposure. Most samples were collected from the East Coast, and the majority of West Coast samples were obtained from actively captured animals, which may have had a lower likelihood of exposure compared with stranded or rehabilitated individuals.

While our study offers valuable insights into the prevalence of SARS-CoV-2 in marine mammals, it is essential to consider some limitations. First, the majority of samples tested were collected opportunistically and consisted primarily of swab specimens for the detection of active SARS-CoV-2 infection. Serum samples, which are more likely to provide evidence of exposure to SARS-CoV-2, were collected from only six of the 21 species from which swabs were collected. Additionally, the sensitivity of RT-PCR assays relies on primer binding to conserved genomic regions, and mutations in SARS-CoV-2 primer target sites can reduce assay sensitivity and potentially result in false negatives. However, the ORF1b region (encoding non-structural protein 14) targeted in our assay is among the most conserved regions of the SARS-CoV-2 genome, which reduces the likelihood that sequence variation substantially affected detection sensitivity [38]. Importantly, in this study, no pan coronavirus or consensus coronavirus PCR assays were performed; therefore, the presence of other coronaviruses in the sampled animals cannot be excluded. However, to date, only a limited number of coronaviruses have been described in marine mammals, primarily alphacoronaviruses and gammacoronaviruses identified in seals and cetaceans [16,17]. Second, sampling for certain species was limited to fewer than ten individuals. Therefore, we may lack information on potential exposure to the virus for many marine mammal species. Third, most serum samples exhibited low to moderate neutralizing titers (≤1:32) despite showing high optical density values in the ELISA, and three confirmatory ELISA-positive samples collected between 2023 and 2024 lacked detectable neutralizing activity. ELISA detects binding antibodies against viral antigens, indicating prior exposure to the virus. However, not all binding antibodies possess neutralizing activity. Therefore, the presence of binding antibodies in the absence of detectable neutralization may reflect exposure that elicited predominantly non-neutralizing antibodies [39]. Although we cannot completely rule out potential cross-reactivity with other coronaviruses, current evidence indicates that ELISA assays targeting the SARS-CoV-2 receptor-binding domain (RBD) are highly specific [40]. In addition, ELISA-positive samples were confirmed using virus neutralization tests (VNT), which further reduces the likelihood of cross-reactivity. However, VNT was performed using only a single SARS-CoV-2 strain (Wuhan-like strain USA/WA1/2020). Given that the sampling period spanned 2020–2025, the inclusion of multiple SARS-CoV-2 strains in the neutralization assay could have increased its sensitivity, particularly for detecting antibodies against antigenically divergent variants. Finally, although the secondary antibody used in the ELISA recognizes immunoglobulins across a broad range of mammalian species and was validated in-house for multiple taxa, including marine mammals (S1 Fig), the possibility of false-negative results, particularly in animals with low antibody titers, cannot be entirely excluded

In conclusion, this survey found no evidence of active SARS-CoV-2 transmission among marine mammals in the United States from 2020 to 2025. Nonetheless, the establishment of endemic infection in a mammalian host with substantial human contact could facilitate viral evolution and spillover as a newly emerging human variant. Although no active infections were found, indirect evidence of past SARS-CoV-2 exposure highlights the importance of future studies on the ecological and molecular mechanisms of SARS-CoV-2 transmission to marine mammals and their responses. It also emphasizes the need for ongoing surveillance of populations that could be at risk.

## Supporting information

S1 Table Metadata for each animal sampled in this study. (XLSX)

S2 Table β-actin detection in swabs collected from marine mammals. Numbers report individual animals for each species as negative (N) or positive (P) for endogenous β-actin detection, Ct < 40. Animals were considered to have viable RNA on swab samples if a minimum of 1 swab sample was positive. Percentage shows the number of animals for each species with viable RNA.
(DOCX)

S1 Fig Species and sample breadth results for ELISA SARS-CoV-2 RBD ELISA. Phylogenetic tree shows IgG1 CH2-CH3 orthologs for representative mammalian orders. Circles denote sample types tested per species: red (serum), blue (oral/nasal), brown (fecal). Circles with an X were tested but failed at the internal control. No circle means a sample could not be collected from that species.
(TIF)

S2 Fig Representative ELISA-RBD plate layout. 96-well Immulon 4 HBX microtiter plate showing the experimental setup and distribution of controls and samples. Black boxes indicate blank wells used for background correction. Red boxes indicate positive controls, and green boxes indicate negative controls. The remaining wells correspond to samples.
(TIF)
