# Supplementary material for: Serological evidence of SARS-CoV-2 exposure in marine mammals in the United States between 2020 and 2025
Source: PLoS One. 2026 Jul 9;21(7):e0351734. doi: 10.1371/journal.pone.0351734 (PMC13349194; doi:10.1371/journal.pone.0351734)
Supplement: S2 Table — Numbers report individual animals for each species as negative (N) or positive (P) for endogenous β-actin detection, Ct < 40. Animals were considered to have viable RNA on swab samples if a minimum of 1 swab sample was positive. Percentage shows the number of animals for each species with viable RNA. (DOCX) [file pone.0351734.s002.docx]

**S2 Table.** **β-actin detection in swabs collected from marine mammals**

|  | Species | β-actin | | | |
| --- | --- | --- | --- | --- | --- |
|  |  | N | P | total | %viable samples |
| Pinnipeds | CA sea lion | 29 | 5 | 34 | 15% |
|  | Grey seal | 47 | 282 | 329 | 86% |
|  | Harbor seal | 31 | 200 | 231 | 87% |
|  | Harp seal | 13 | 50 | 63 | 79% |
|  | Hawaiian monk seal | 0 | 8 | 8 | 100% |
|  | Hooded seal | 0 | 3 | 3 | 100% |
|  | Northern elephant seal | 20 | 3 | 23 | 13% |
|  | Northern fur seal | 0 | 8 | 8 | 100% |
|  | Pacific walrus | 0 | 2 | 2 | 100% |
|  | Ribbon seal | 3 | 8 | 11 | 73% |
|  | Spotted seal | 2 | 12 | 14 | 86% |
|  | Steller sea lion | 53 | 26 | 79 | 33% |
| Cetaceans | Beaked whale |  | 1 | 1 | 100% |
|  | Minke whale |  | 1 | 1 | 100% |
|  | Beluga whale |  | 4 | 4 | 100% |
|  | Common dolphin |  | 7 | 7 | 100% |
|  | Harbor porpoise |  | 7 | 7 | 100% |
|  | Humpback whale |  | 2 | 2 | 100% |
|  | Spotted dolphin | 1 | 0 | 1 | 0% |
|  | Striped dolphin | 1 | 1 | 2 | 50% |
|  | White sided dolphin |  | 2 | 2 | 100% |
| Mustelids | Sea otter | 2 | 24 | 26 | 92% |
