# Supplementary material for: Serological evidence of SARS-CoV-2 exposure in marine mammals in the United States between 2020 and 2025
Source: PLoS One. 2026 Jul 9;21(7):e0351734. doi: 10.1371/journal.pone.0351734 (PMC13349194; doi:10.1371/journal.pone.0351734)
Supplement: Attachment — Submitted filename: Response to Reviewers.pdf [file pone.0351734.s006.pdf]

Comments from Academic Editor

1. Please ensure that your manuscript meets PLOS ONE's style requirements, including those for file naming. The PLOS ONE style templates can be found at

https://journals.plos.org/plosone/s/file?id=wjVg/PLOSOne_formatting_sample_main_body.pdf
and

https://journals.plos.org/plosone/s/file?id=ba62/PLOSOne_formatting_sample_title_authors_affiliations.pdf

Response: *We have revised the manuscript to ensure that it complies with the style requirements of PLOS ONE.*

2. To comply with PLOS One submissions requirements, in your Methods section, please provide additional information regarding the experiments involving animals and ensure you have included details on (1) methods of sacrifice, (2) methods of anesthesia and/or analgesia, and (3) efforts to alleviate suffering.

Response: *Thank you for this comment.*

No animals were sacrificed or experimentally manipulated for the purposes of this study. Samples were obtained opportunistically from animals admitted to rehabilitation centers, from stranded animals found dead, and from animals captured and released as part of monitoring programs, during which biological samples were collected. ***We did not perform any procedures involving anesthesia or euthanasia. All animal handling was performed by trained marine mammal rehabilitation personnel following standard animal welfare protocols****. This information has now been clarified in the Methods section on* ***page 6, lines 129 - 131****.*

3. We note that Figure 2 in your submission contain [map/satellite] images which may be copyrighted. All PLOS content is published under the Creative Commons Attribution License (CC BY 4.0), which means that the manuscript, images, and Supporting Information files will be freely available online, and any third party is permitted to access, download, copy, distribute, and use these materials in any way, even commercially, with proper attribution. For these reasons, we cannot publish previously copyrighted maps or satellite images created using proprietary data, such as Google software (Google Maps, Street View, and Earth). For more information, see our copyright guidelines: http://journals.plos.org/plosone/s/licenses-and-copyright.

Response: *Thank you for highlighting this point. The base map used in Figure 2 was obtained from the U.S. Census Bureau (https://www.census.gov/) and is in the public domain. We have now added the following statement to the Figure 2 caption (****page 12, lines 255 – 256****):* ***"State boundaries were derived from public domain U.S. Census Bureau TIGER/Line shapefiles"****. The corresponding citation has also been included in the reference list (****Reference [26]****).*

If the reviewer comments include a recommendation to cite specific previously published works, please review and evaluate these publications to determine whether they are relevant and should be cited. There is no requirement to cite these works unless the editor has indicated otherwise

Comments from Reviewer #1

Reviewer #1: The manuscript "Serological Evidence of SARS-CoV-2 Exposure in Marine Mammals in the United States between 2020 and 2025" is an interesting manuscript that reports evidence of SARS-CoV-2 exposure in marine mammals. The information presented by the authors is interesting and important to understand the ecology of SARS-CoV-2 and is clear that was a really big endeavor to achieve. However, despite the importance of this report, there are many technical issues that must be solved before being considered to be published. Also lab results interpretation is limited and as a consequence discussion is limited and for moments contradictory.

The main methodology issue is the performance of the ELISA. Authors do not describe in detail how this was performed. The reference that they present to validate the use of this test is for human test, not for a panmammalian as is claimed in the manuscript.

To properly evaluate the results detailed methodology is needed to detect possible pitfalls in the laboratory analyses. One of the most important details is the secondary antibody or protein used, as well as, all the controls used.

Response*: Thank you for this comment. We have expanded the ELISA methodology in the revised manuscript to provide additional experimental details and clarify the controls used. Although the SARS-CoV-2 ELISA was initially developed to detect antibodies against the receptor-binding domain (RBD) of SARS-CoV-2 in human sera* (ref: https://doi.org/10.1002/cpmc.100)*, the assay was previously adapted and optimized in our laboratory for the detection of SARS-CoV-2 antibodies in ferret samples* (ref: https://doi.org/10.1073/pnas.2025601118). *Before this study, the ELISA assay was also validated for detecting IgG antibodies in serum samples from multiple mammalian orders, including marine mammals. These validation data are now provided in Supplementary Figure 1 and are referenced in the Methods section (**page 8, lines 159 – 160**).*

*To enable antibody detection across mammalian species, we used Pierce Recombinant Protein A/G Peroxidase Conjugate as the secondary antibody, and this information was added to the Methods section (**page 8, line 168**). This fusion protein combines the IgG-binding domains of Protein A and Protein G and binds the Fc region of IgG antibodies from a broad range of mammalian species, making it suitable for multi-species serological studies.*

*As a positive control, we used serum from spike (S) protein-immunized alpacas, as marine mammal sera with confirmed SARS-CoV-2 antibodies were not available. As negative controls, we used pre-pandemic gray seal sera collected during avian influenza monitoring programs in 2018. This information is provided in the Methods section (**page 8, lines 174-175**).*

About the interpretation of the lab results, it is necessary to declare the limitation of the test used. For example, how specific are the primers used for the rt-pcr? If the primers are so specific and the strain is little different, the rt-pcr can lose the diagnostic, otherwise, a more degenerated primer can detect even different variants of the virus, making the result much more trustful.

***Response**: Thank you for this comment. The RT-PCR assay used in this study targeted the ORF1b (NSP14) region of SARS-CoV-2, which is highly conserved among SARS-CoV-2 lineages and has been widely used for sensitive detection of the virus. In addition, β-actin was amplified as an internal control to confirm RNA integrity and sample quality.*

We acknowledge that the sensitivity of RT-PCR assays relies on primer binding to conserved genomic regions, and mutations in SARS-CoV-2 primer target sites can reduce assay sensitivity and potentially result in false negatives. However, the ORF1b region (encoding non-structural protein 14) targeted in our assay is among the most conserved regions of the SARS-CoV-2 genome, which reduces the likelihood that sequence variation substantially affected detection sensitivity (https://pmc.ncbi.nlm.nih.gov/articles/PMC9933857/). *This limitation has now been included in the Discussion section on **page 15, lines 326-330**.*

Some data of the animals is missing. This is especially important for Zalophus, where the animals tested positive where actively captured. The site of capture is vital to understand how the animals get in contact with the virus.

Response**: In this study, we collaborated with more than 22 marine mammal rehabilitation centers as well as personnel from the National Oceanic and Atmospheric Administration (NOAA). Because samples were collected through multiple agencies and under varying field conditions and onsite protocols, metadata associated with individual animals varies across sample sources. However, to address the reviewer's concern, we have added information in the Discussion section describing the geographic context in which California sea lions were sampled, which may help interpret potential exposure to the virus. The following sentence has been added to the Discussion section as follows (**page 14, Lines 306-309**): **Moreover, the California sea lions sampled in this study were captured and released in San Miguel Island, where they may interact with humans through activities such as ecotourism and research operations. These human-animal interfaces may increase opportunities for exposure to SARS-CoV-2 through spillover from infected individuals.

Discussion should be rewrite and reinforce, in its actual form is just a superficial comparison. One important issue to be discussed is the lab results (based on the methodology used) and its limitations. The discussion is lack of analysis of the geographic distribution, difference of the species, antibody titer, among others.

Response*: Thank you for this valuable comment. We have substantially revised and expanded the Discussion to strengthen the interpretation of our findings. Specifically, we now provide a more detailed analysis of the serological results in the context of the methodology used, including the strengths and limitations of the ELISA and virus neutralization assays (****page 15, lines 326-351)****. We also expanded the discussion to better examine patterns in the data, including geographic distribution of seropositive animals, differences among species, and variation in antibody titers. In addition, we incorporated relevant examples from rehabilitation histories and ecological contexts that may help explain potential exposure to SARS-CoV-2 (****page 14, lines 301-320****).*

Here some specific comments that could help to improve the manuscript:

Line 84-85: this sentence does not have enough sustain. The similitude does not explain why could be potentially infected at "low viral concentration", is it because it is similar to humans and humans can infect a low viral concentration?

Response*: Marine mammal species were predicted to be susceptible to SARS-CoV-2 based on in silico modeling of the binding affinity between the viral spike protein and the cellular receptor ACE2, using human ACE2 as a reference. We intended to emphasize that certain marine mammal species may be highly susceptible to SARS-CoV-2 infection based on those studies. However, we agree with the reviewer that the original sentence could lead to confusion. To improve clarity, we removed the sentence "the similarity level of their ACE2 receptor with human ACE2, indicating SARS-CoV-2 infection could potentially be caused by a low viral concentration. The revised text now reads as follows (****page 4, lines 85–87****):* ***Numerous marine mammal species, including most pinniped and cetacean species and particularly harbor seals, were predicted to be highly susceptible to SARS-CoV-2 infection based on in silico modeling of SARS-CoV-2 spike protein binding affinity to their ACE2 receptors.***

Line 124: viral transport mediums need specification to know how long the virus or at least the virus RNA can last.

Response*: The composition of the viral transport medium has been specified in the Methods section (****page 6, line 134-135****).*

Line 135: declare the primers used and the range of detection for SARS-CoV-2. How specific are the primers just a specific strain of any SARS-CoV-2 strain.

***Response**: All samples were tested for SARS-CoV-2 viral RNA using a real-time RT-qPCR assay targeting the open reading frame 1b non-structural protein 14 (ORF1b-nsp14) region, using the HKU-ORF1 primer–probe set described by Chu et al. [23] (Forward 5'-TGGGGYTTTACRGGTAACCT-3', Reverse 5'-AACRCGCTTAACAAAGCACTC-3', Probe 5'-FAM-TAGTTGTGATGCWATCATGACTAG-TAMRA-3'). This assay targets a highly conserved region of the SARS-CoV-2 genome and is designed to detect diverse SARS-CoV-2 lineages rather than a specific strain. We have specified the primer–probe set used for SARS-CoV-2 detection in the Methods section (**page 7, lines 146-148**).*

Line 143: the 23 reference does not include a generic protocol for mammals, this article is about tests for humans.

***Response**: As noted above, the ELISA protocol implemented in this study was previously applied by K. Sawatzki et al. (https://www.pnas.org/doi/10.1073/pnas.2025601118) to detect SARS-CoV-2 antibodies in ferrets, demonstrating its applicability to non-human mammalian samples. Prior to this study, the assay was validated in-house to detect IgG from a wide range of mammals, including marine mammals. To address the reviewer's concern and improve clarity and reproducibility, we have expanded the methodological description of the ELISA protocol in the Methods section (**page 8, lines 159 – 160**) and added Supporting Information Figure S1 to illustrate IgG detection across several species.*

Line 144-145: Authors omitted the most important part of the protocol, since the previous reference is for testing in humans.

***Response**: The ELISA protocol has been expanded to include additional methodological details (**page 8, lines 159-170**)*

Clearly and highly detailed protocol must be included, with emphasis in secondary antibody. The secondary antibody (or protein) must be capable of recognizing FC from immunoglobulins of mammals. This part is crucial to understand the effectiveness of the serological test.

***Response**: Information regarding the secondary antibody, specifically the Pierce Recombinant Protein A/G Peroxidase Conjugate, has now been included in the Methods section (**page 8, line 168**).*

If there is not a previous use of this methodology, authors must include the standardization phase as a supplementary material.

***Response**: The ELISA protocol implemented in this study was previously adapted and used by K. Sawatzki et al.* (https://www.pnas.org/doi/10.1073/pnas.2025601118) *to detect SARS-CoV-2 antibodies in ferrets. However, as noted above, the ELISA assay was validated in-house for detecting IgG antibodies in serum samples from several wildlife mammalian species. The validation data are shown in Supplementary Figure 1 and referenced in the Methods section (**page 8, lines 159 – 160**).*

Line 147: what the authors used has negative controls?

***Response**: Pre-pandemic serum samples collected from gray seals in 2018 during influenza monitoring programs were used as negative controls, allowing us to establish the background signal and support the specificity of the ELISA assay. This information is provided in the Methods section (**page 8, line 175**).*

Line 149: What does "healthy" mean for the authors? How could the authors rule-out a subclinical or previous exposition?

***Response**: We agree with the reviewer that subclinical exposure cannot be completely ruled out. We intended to indicate that these serum samples were collected before the emergence of SARS-CoV-2, making prior exposure to this virus highly unlikely. To avoid ambiguity, the term "healthy" (**page 8, line 175**) has been removed from the manuscript.*

Line 149: why the authors selected this species and no others?

Response: The positive control reagent was kindly provided by our colleague on the Tufts campus, Dr. Charles Shoemaker. Unrelated to the work reported here, Dr. Shoemaker has undertaken studies to develop camelid antibodies against SARS-CoV-2, wherein alpacas were immunized with the spike (S) protein and developed robust antibody responses to the S protein.

Line 156: If a validation (or more precisely comparison) is attempted, a statistical test must be run, such as a concordance test such as kappa. And in this way, positive and negative samples must be included, not just positive.

***Response**: Thank you for this comment. We agree that statistical concordance analyses, such as Cohen's kappa, are appropriate for formally comparing the diagnostic performance of two assays and require both positive and negative samples. In the present study, however, the neutralization assay was not used in comparison to the ELISA, but rather to confirm the presence of functional*

neutralizing antibodies in ELISA-positive samples. Therefore, only ELISA-positive sera were selected for neutralization testing. Because ELISA-negative samples were not included in this analysis, a concordance test, such as kappa, could not be performed.

Line 163: Controls for the neutralization test must be declared. How the authors can test the true inactivation of the serum. Some carnivore serums are still killing cells after heat (56oC) inactivation.

***Response**: Each plate included a virus-only control, a cell-only control, and a serum-only control, and this information has been added to the Methods section (**page 9, lines 186-187**). Serum cytotoxicity was assessed by incubating heat-inactivated sera with cells in the absence of virus.*

Line 165: a data analyses section is necessary

***Response**: We have added a statistical analysis section to the Methods (**page 9, lines 192-196**).*

Line 172: authors can not ensure if the virus is active just by pcr. But you can say that at least there is some RNA of the virus in the sample.

*Response: We agree with the reviewer. The sentence has been revised and reads as follows (**page 10, line 206)**: "All swabs were negative for SARS-CoV-2 RNA (Table S1), indicating the absence of detectable SARS-CoV-2 RNA in marine mammal samples collected from 2020 to 2025."*

Line 174: even when authors already mention how many samples analyzed, it would be helpful to put 18 out of how many, and of course a prevalence or frequency along with confidence intervals.

***Response**: We thank the reviewer for this suggestion. The Results section has been revised to include the total number of samples analyzed, along with the corresponding prevalence and 95% confidence intervals (**page 10, line 208**).*

Line 175: since CA sea lions come from active capture, it is important to declare and to discuss where the animals were captured. Especially since this species is more likely to have a natural infection. Is this site close to human settlements?

***Response**: We have addressed this comment above. We have included information in the Discussion section regarding the sampling locations of California sea lions (**page 14, lines 306-309**).*

Line 178: Same 12 samples of the confirmatory ELISA?
***Response**: No. Due to limited sample volumes, not all samples were tested by the Spike ELISA; some were reserved for the virus neutralization test (VNT). Of the 12 ELISA spike-positive samples, nine were confirmed positive by VNT, and three additional samples, including two not tested by Spike ELISA and one Spike ELISA-negative sample, were also positive. This information has been added to the Results section (**page 10, lines 212-213)**.*

Line 180: the first sentence is confusing, it looks like authors test both condition stranded and active capture. However, just samples from grey came from both sources, active and stranded, so this declaration implies that you evaluate both.

***Response**: We agree that the original sentence may have been confusing. The text has been revised for clarity and now reads as follows (**page 10, lines 215-217**): **Seropositive samples were detected in harbor seals, gray seals, harp seals, and California sea lions. In gray seals, no neutralizing antibodies were detected in samples collected during capture–release studies**.*

Line 187: this raises some questions. Samples were analyzed separately? This is increasing the number of positive samples. Authors must clean the data base specially of the resampling individuals where there is not so much time difference.

***Response**: The animal was included only once in the dataset and therefore does not inflate the number of positive samples. However, we reported the resampling information because the animal tested positive on two separate occasions, which we considered important to document.*

Line 191: is not 3/7 since one was not tested, must be 3/6

***Response**: We agree with the reviewer. This was an error and has now been corrected in the manuscript (**page 10, line 227**).*

Line 195-197: some of the table legend was already in the methodology, not necessary here.

***Response**: We have removed the redundant information from the table legend.*

Line 199: is necessary to include confidence interval

***Response**: We have included 95% confidence intervals for all reported proportions throughout the manuscript.*

Line 201: Is important to mention that you are considering a positive animal (or sample) when it was positive to ELISA and SN.

*Response: We have clarified that an animal was considered seropositive only if the sample tested positive by both ELISA and VNT (**page 9, lines 190-191**).*

Line 202-205: Authors have data to compare prevalence between species, maybe sex and age, why the authors did not? A simple x-square test can solve this. It was a big effort to lose this information.

***Response**: Differences in seroprevalence among species were assessed using Fisher's exact test, which is appropriate given the small sample sizes (**page 9, line 195)**. Data on sex and age were not included because this information was unavailable for the majority of samples.*

Line 229: the specific clinical history of the positive animals, specially from those stranded, are really important to evaluate the odds of get infected in the rehabilitation center. Antibodies usually need at least 21 to get formed, in this way if the sample was get before that time, seroconversion should be before the contact to humans.

***Response**: As noted above, metadata associated with individual animals were not consistently available across all samples. Nevertheless, to address the reviewer's concern, we have added a brief clinical history of a seropositive harbor seal with high neutralizing titers to the discussion section, highlighting circumstances that may have provided opportunities for human-to-animal SARS-CoV-2 transmission (**page 13, lines 282-287**).*

Line 250-252: Make clear what is the relevance of this for a SARS-CoV-2 study, particularly the final part of this paragraph "the clinical significance of the HSCoV remains unclear due to incomplete sampling during the outbreak"

Response**: We intended to highlight that coronaviruses can infect marine mammals and may be associated with disease outbreaks. We added the following sentence to the Discussion section (**page 14, lines 298-300)**: **Nevertheless, this finding demonstrates that coronaviruses can infect marine mammals and may be associated with disease outbreaks.

Line 255-257: No agree with this, new variants are more specific to humans, original ones had more potential to cross transition.

Response**: We agree that simply stating that RBD mutations increase cross-species transmission potential is broad. While certain RBD substitutions enhance binding to human ACE2, host range is determined by multiple factors. Accordingly, we have revised the sentence to read (**page 14, lines 303-306**): **However, while in silico predictions provide useful insight into receptor compatibility, SARS-CoV-2 has demonstrated the capacity to infect a broad range of mammalian hosts, indicating that factors beyond ACE2 binding affinity contribute to host susceptibility.

Line 274: "using contemporary SARS-CoV-2 strains in the VNT could have increased its sensitivity" indeed but also if the antibodies of your study came from cross reaction, new strain could detect even lower prevalence.

Response**: We agree with the authors that antibodies resulting from cross-reactive responses could decrease the sensitivity of the VNT. However, we want to emphasize that including multiple SARS-CoV-2 strains might improve the assay's sensitivity by detecting a wider range of neutralizing antibody responses. We have now changed the sentence, and it reads as follows (**page 15, lines 346-349**): **Given that the sampling period spanned 2020 - 2025, the inclusion of multiple SARS-CoV-2 strains in the neutralization assay could have increased its sensitivity, particularly for detecting antibodies against antigenically divergent variants.

Figures: quality must be improved.

***Response**: We have validated the quality of all figures using the PLOS ONE free figure-checking tool, NASS.*

I recommend to the authors to explore to the natural dynamics of immunoglobulin production and decay. Also, difference between neutralizing antibodies and those antibodies that have not neutralizing activity. To considered exposure to the virus, this difference is important. In this way, almost all antibodies need exposure to the antigen to be produce, but not necessary all produce neutralizing activity (i.e. protection to the pathogen).

Response**: We thank the reviewer for this insightful suggestion. We agree that the dynamics of immunoglobulin production and decay, as well as the distinction between binding antibodies and functional neutralizing antibodies, are important considerations when interpreting serological data. We have now clarified this distinction and its implications for interpreting exposure in the Discussion section (**page 15, lines 339-342**): **ELISA detects binding antibodies against viral antigens, indicating prior exposure to the virus. However, not all binding antibodies possess neutralizing activity. Therefore, the presence of binding antibodies in the absence of detectable neutralization may reflect exposure that elicited predominantly non-neutralizing antibodies.

It is important to know if your secondary antibody allows you to identify the isotype of immunoglobulin that you found in the ELISA test. Is not the same interpretation if you find IgG or IgM.

***Response**: We thank the reviewer for this important comment. As noted above, the secondary antibody used was Pierce Recombinant Peroxidase Conjugated Protein A/G, which is designed to detect antibodies, particularly IgG isotypes, from a broad range of species. Prior to this study, we validated in-house that this secondary antibody effectively detects IgG antibodies from a wide range of wildlife species, including marine mammals. This information is now provided in the supporting information (S1 Fig) and referenced in the Methods section (**page 8, lines 159 – 160**).*

Finally, the description of other potential coronaviruses present in marine mammals is important and even more, how probable is the cross reaction with SARS-CoV-2. This is a key point since there are no other complementary techniques.

***Response**: Although we cannot completely rule out the possibility of cross-reactivity with other coronaviruses, current evidence indicates that ELISA assays targeting the SARS-CoV-2 receptor-binding domain (RBD) are highly specific. Furthermore, all ELISA-positive samples were subsequently confirmed by virus neutralization testing (VNT), increasing the robustness of our serological findings. To date, only a limited number of coronaviruses have been described in marine mammals, primarily alphacoronaviruses and gammacoronaviruses identified in seals and cetaceans, and no betacoronaviruses closely related to SARS-CoV-2 have been reported in these hosts. These points have now been incorporated into the Introduction and Discussion sections (**page 4, lines 91-95** and **page 15, lines 333-334**, respectively).*

Comments from Reviewer #2

Reviewer #2: Overall, this article is well written and presents interesting findings. However, several methodological and interpretive points require clarification to strengthen confidence in the results.

General Comments

The authors do not mention whether any pan coronavirus or consensus coronavirus PCR assays were performed on the swabs. Given the possibility of cross reactivity in serological assays, this information is important for interpreting the results.

***Response**: We thank the reviewer for this comment. In this study, we did not perform pan-coronavirus or consensus-coronavirus PCR assays on the swab samples. Our molecular screening specifically targeted the SARS-CoV-2 ORF1b region using a validated RT PCR assay. We acknowledge that testing for other coronaviruses could provide additional information for interpreting potential cross-reactivity in serological assays. However, the ELISA assay used in this*

*study targets the SARS-CoV-2 Spike receptor-binding domain, and positive samples were confirmed by virus neutralization tests, which are highly specific and minimize the risk of cross-reactivity. In addition, to date, only a limited number of coronaviruses have been described in marine mammals, primarily alphacoronaviruses and gammacoronaviruses identified in seals and cetaceans, and no betacoronaviruses closely related to SARS-CoV-2 have been reported in these hosts. We have included these points in the Introduction and Discussion sections (**page 4, lines 91-95** and **page 15, lines 333-334**, respectively).*

Because serological assays can yield false positives, the study design and conclusions should be interpreted cautiously. The authors should report the sensitivity of the ELISA RBD assay particularly in any relevant mammalian species to help readers assess the likelihood of missed positives.

***Response**: This comment has been addressed below (**Line-specific comments 142–144**)*

Virus neutralization testing (VNT) was not performed on all serum samples. The authors should clarify how they calculated the percentage of neutralizing antibodies and whether these analyses are appropriate given that VNT was only conducted on indirect ELISA RBD–positive samples.

***Response**: This comment has been addressed below (**Line-specific comments 198-201 and 204-205**)*

Including a larger number of pre pandemic serum samples would strengthen the study by helping determine whether the assays produce any unexpected positives and by increasing confidence in the VNT results.

***Response**: This comment has been addressed below (**Line-specific comments 149-150**).*

The absence of evidence for active infection makes it difficult to interpret the low to moderate VNT titers observed in a few animals. Additionally, different VNT formats can vary in false positive and false negative rates, which should be acknowledged.

*Response: Thank you for this comment. We agree that the absence of detectable active infection limits the interpretation of the low to moderate neutralizing titers observed in some animals. We have added statements to the Discussion (**page 15, lines 339-349**) acknowledging that VNT results may be influenced by cross-reactive antibodies or low antibody titers.*

Line Specific Comments

Lines 142–144: What is the sensitivity of the ELISA RBD assay, particularly for any relevant mammalian species? Including this information would help readers assess the likelihood of false negatives. If sensitivity is low, some positive animals may have been missed; if high, it strengthens confidence in the findings.

*Response: Although the RBD-based ELISA used in this study is considered highly sensitive, we cannot completely exclude the possibility of false negatives. However, the secondary antibody used in the ELISA recognizes immunoglobulins across a broad range of mammalian species and was validated in-house for multiple taxa, including marine mammals. This limitation has now been acknowledged in the manuscript to facilitate interpretation of the serological results (**page 15, lines 349-351**). In addition, Supporting Information S1 Fig has been included to illustrate IgG detection across several wildlife mammalian species.*

Lines 149–150: Only one pre pandemic serum sample was used as a negative control. Is it possible to include additional pre pandemic samples? This would help determine whether any of the assays produce unexpected positives and would strengthen the overall conclusions.

***Response**: We thank the reviewer for raising this point. There was a lack of clarity in the description of the ELISA protocol. In fact, 8 pre-pandemic serum samples were used as negative controls in the ELISA assays as mentioned in the Methods section (**page 8, lines 173-175**).*

Line 156: If I understand correctly, all serum samples were first screened using the ELISA RBD assay, and only RBD positive samples were subsequently tested by ELISA Spike and VNT. If this is accurate, please clarify the reference to “20 positive samples (n = 20)” here. In the Results section, you report 18 ELISA RBD positive samples. Reconciling these numbers would improve clarity.

***Response**: We thank the reviewer for noting this discrepancy. This was an error in the manuscript, and the text has now been corrected to ensure consistency with the reported number of ELISA RBD–positive samples (**page 9, line 181)**.*

Lines 166–168: Please specify in the results section the number of animals tested by PCR and by ELISA. Although the table provides totals, including these figures directly in the text would improve readability. For example, briefly state the total number of animals tested, how many underwent PCR, how many were tested serologically, and how many received both.

*Response: We thank the reviewer for this suggestion. The Results section has been revised to report the number of animals tested by PCR, by serology, and by both. We included the following sentence on **page 9, lines 198-199: A total of 1,017 individual animals were tested. Among these, 639 were tested only for SARS-CoV-2 RNA, 163 were tested only for antibodies, and 215 were tested for both viral RNA and antibodies.***

Lines 198–201: As I understand it, VNT was performed only on ELISA-RBD positive samples. If so, analyses comparing VNT results across ELISA RBD negative animals may not be appropriate unless VNT was performed on all samples or the ELISA RBD assay is known to be 100% sensitive.

Lines 204–205: The same concern applies here: analyses involving VNT results should account for the fact that VNT was not conducted on ELISA RBD negative samples.

Response*: In this study, the ELISA RBD, which is known to be highly specific, was used as a screening assay to identify presumptive positive samples, and all the samples were tested in duplicate. Only ELISA-RBD–positive samples were subsequently tested using virus neutralization testing (VNT) for confirmation. We have clarified in the manuscript (****page 9, lines 190-191****) that an animal was considered seropositive only if the corresponding sample tested positive by both ELISA and VNT. Therefore, VNT results were not intended to be compared across ELISA-negative samples; rather, VNT was used solely to confirm seropositivity among samples identified as positive by the screening ELISA.*

---

6. PLOS authors have the option to publish the peer review history of their article (what does this mean?). If published, this will include your full peer review and any attached files.

If you choose "no", your identity will remain anonymous but your review may still be made public.

Do you want your identity to be public for this peer review? For information about this choice, including consent withdrawal, please see our Privacy Policy.

Reviewer #1: No

Reviewer #2: No

[NOTE: If reviewer comments were submitted as an attachment file, they will be attached to this email and accessible via the submission site. Please log into your account, locate the manuscript record, and check for the action link "View Attachments". If this link does not appear, there are no attachment files.]

To ensure your figures meet our technical requirements, please review our figure guidelines: https://journals.plos.org/plosone/s/figures

You may also use PLOS's free figure tool, NAAS, to help you prepare publication quality figures: https://journals.plos.org/plosone/s/figures#loc-tools-for-figure-preparation.

NAAS will assess whether your figures meet our technical requirements by comparing each figure against our figure specifications.

Response*: Both images have been assessed with NAAS and comply with specifications.*
