# Supplementary material for: Serological evidence of SARS-CoV-2 exposure in marine mammals in the United States between 2020 and 2025
Source: PLoS One. 2026 Jul 9;21(7):e0351734. doi: 10.1371/journal.pone.0351734 (PMC13349194; doi:10.1371/journal.pone.0351734)
Supplement: Attachment — Submitted filename: Response to Reviewers_R2.pdf [file pone.0351734.s008.pdf]

Comments from Academic Editor

There are minor edits which should addressed prior to publication of this manuscript. Thank you for your detailed responses to the reviewers' comments.

Response: *We have addressed the reviewers' comments below.*

Comments from Reviewer #1

Reviewer #1: Authors solve all queries. Just a few minor comments.

Response: *Thank you!*

It will be desirable to include representative figures of the controls, positives and negatives samples of the seroneutralization test.

Response: *We thank the reviewer for this helpful suggestion and agree that inclusion of representative images would enhance the presentation of the assay. However, representative images from the seroneutralization (SN) assay were not systematically recorded at the time of the experiments and therefore cannot be provided retrospectively. To nevertheless provide a visual representation of the samples' serological profiles, we have included an image of the corresponding ELISA plate in the Supplementary Material (Fig. S2). For reference, we also provide below a representative image of the SN assays in our lab.*

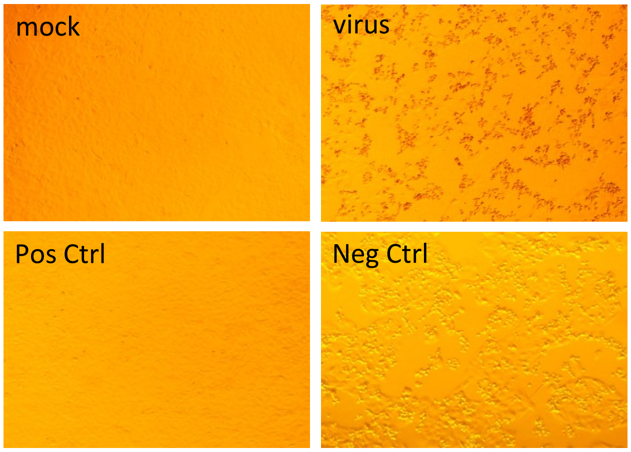

The above images are provided for Reviewers as a reference point in regards to the question of example images. We do not routinely image each well of the neutralization assays. Each well is viewed through the light scope and tallied as neutralized or not neutralized on a plate map template.

*In addition, SN results were independently evaluated by two operators, and this has now been explicitly stated in the Methods section (**line 184**) to strengthen confidence in the scoring procedure.*

Protein A/G binds to the FC of any immunoglobulin, is not specific for IgG, therefore authors are reporting total Ig.

Inganäs, M. (1981). Comparison of Mechanisms of Interaction between Protein A from Staphylococcus aureus and Human Monoclonal IgG, IgA and IgM in Relation to the Classical Fcγ and the Alternative F (ab') 2γ Protein A Interactions. Scandinavian journal of immunology, 13(4), 343-352.

Response:

*We agree with the reviewer that Protein A/G may bind multiple immunoglobulin classes and is not strictly IgG-specific. In response, we have revised the manuscript at **line 154** to clarify that our assay detects total immunoglobulins rather than IgG specifically.*

Scientific names should be used more than common names, once both names were mentioned.

Response*: We thank the reviewer for this suggestion and agree that consistent use of scientific names improves clarity and precision. We have revised the manuscript to preferentially use scientific names after their first mention alongside common names.*

Comments from Reviewer #2

All comments have been addressed.

Response: *Thank you!*
