# Supplementary material for: Serological evidence of SARS-CoV-2 exposure in marine mammals in the United States between 2020 and 2025
Source: PLoS One. 2026 Jul 9;21(7):e0351734. doi: 10.1371/journal.pone.0351734 (PMC13349194; doi:10.1371/journal.pone.0351734)
Supplement: Attachment — Submitted filename: Comments-PLOs One-PONE-D-26-02880_R2. Reviewer 2.pdf [file pone.0351734.s007.pdf]

Serological Evidence of SARS-CoV-2 Exposure in Marine Mammals in the United States between 2020 and 2025

Revision Number 2

Ruba Abdulrahman Ashy

Comments to the Authors:

Reviewer comment #1

1. Abstract and Introduction

The Introduction is well written and provides a good background on SARS-CoV-2 spillover and marine mammal susceptibility. However, it does not fully justify the study design, hypothesis, significance of serology, or the interpretation of findings reported in the abstract as follows:

1.1. Aim and hypothesis: To make the study scientifically stronger, the author should sufficiently define the objectives and hypothesis and emphasize that this study is the first to provide serological evidence of SARS-CoV-2 exposure in marine mammals in the United States.

1.2. There is a lack of a clear rationale for combining PCR and serology. As the abstract reports both RT-qPCR and serological testing, the Introduction mainly discusses infection risk. It does not clearly explain why antibody surveillance is essential in addition to PCR testing.

1.3. The abstract emphasizes that this is the "first serological evidence" of SARS-CoV-2 exposure in marine mammals in the United States. However, the Introduction does not adequately explain why serological detection alone is epidemiologically important.
Could the authors explain the significance of detecting neutralizing antibodies in marine mammals despite the absence of active infection by RT-qPCR?

1.4. Additionally, given the gap, the authors should note which surveillance studies have already been conducted globally and which gaps remain.

Reviewer comment #2

2. Methodology

The methodology is strong and suitable for its objectives, with notable strengths in laboratory workflow and confirmatory testing. The study demonstrates excellent adherence to ethical standards. It includes a large, diverse sample and a rigorous serological testing strategy.

Below are some improvements that could be clarified to enhance the study's rigour:

2.1. Have the authors recorded any demographic data for the sampled animals, such as sex, age, and clinical condition, since environmental factors may influence serological findings? Please add demographic and environmental variables.

2.2. According to Table 1, the geographic and temporal distributions of the samples collected between 2020 and 2025 were not included in the table. Could the authors add additional information regarding the sampling locations and yearly distribution of the collected samples, if applicable?

Reviewer comment #3

3. Results

The results are clearly displayed and well structured. There are no major methodological errors.

Reviewer comment #4

4. Discussion

The discussion is well written and has an excellent start emphasizing that this study is the first in investigating SARS-CoV-2 in several marine mammal species on both the East and West coasts of the U.S.

Reviewer comment #5

5. Figures

Figures 1 and 2 need to be prepared in Illustrator to ensure the highest resolution. Alternatively, they can be exported in high-quality vector formats, such as PDF or EPS, which are preferred for publication.
