# Supplementary material for: Serological evidence of SARS-CoV-2 exposure in marine mammals in the United States between 2020 and 2025
Source: PLoS One. 2026 Jul 9;21(7):e0351734. doi: 10.1371/journal.pone.0351734 (PMC13349194; doi:10.1371/journal.pone.0351734)
Supplement: Peer review history

# Peer review: Decision Letter 0

**DOI:** 10.1371/journal.pone.0351734.r001

**Authors:** Stephen Raverty

2 Mar 2026

PONE-D-26-02880

Serological Evidence of SARS-CoV-2 Exposure in Marine Mammals in the United States between 2020 and 2025

PLOS One

Dear Dr. Sanogo,

Thank you for submitting your manuscript to PLOS ONE. After careful consideration, we feel that it has merit but does not fully meet PLOS ONE’s publication criteria as it currently stands. Therefore, we invite you to submit a revised version of the manuscript that addresses the points raised during the review process.

Please submit your revised manuscript by Apr 16 2026 11:59PM. If you will need more time than this to complete your revisions, please reply to this message or contact the journal office at plosone@plos.org. When you're ready to submit your revision, log on to https://www.editorialmanager.com/pone/ and select the 'Submissions Needing Revision' folder to locate your manuscript file.

Please include the following items when submitting your revised manuscript:

- A letter that responds to each point raised by the academic editor and reviewer(s). You should upload this letter as a separate file labeled 'Response to Reviewers'.
- A marked-up copy of your manuscript that highlights changes made to the original version. You should upload this as a separate file labeled 'Revised Manuscript with Track Changes'.
- An unmarked version of your revised paper without tracked changes. You should upload this as a separate file labeled 'Manuscript'.

If you would like to make changes to your financial disclosure, please include your updated statement in your cover letter. Guidelines for resubmitting your figure files are available below the reviewer comments at the end of this letter.

If applicable, we recommend that you deposit your laboratory protocols in protocols.io to enhance the reproducibility of your results. Protocols.io assigns your protocol its own identifier (DOI) so that it can be cited independently in the future. For instructions see: https://journals.plos.org/plosone/s/submission-guidelines#loc-laboratory-protocols. Additionally, PLOS ONE offers an option for publishing peer-reviewed Lab Protocol articles, which describe protocols hosted on protocols.io. Read more information on sharing protocols at https://plos.org/protocols?utm_medium=editorial-email&utm_source=authorletters&utm_campaign=protocols.

We look forward to receiving your revised manuscript.

Kind regards,

Stephen Raverty

Academic Editor

PLOS One

Journal requirements:

When submitting your revision, we need you to address these additional requirements.

1. Please ensure that your manuscript meets PLOS ONE's style requirements, including those for file naming. The PLOS ONE style templates can be found at

https://journals.plos.org/plosone/s/file?id=wjVg/PLOSOne_formatting_sample_main_body.pdf and

https://journals.plos.org/plosone/s/file?id=ba62/PLOSOne_formatting_sample_title_authors_affiliations.pdf

2. To comply with PLOS One submissions requirements, in your Methods section, please provide additional information regarding the experiments involving animals and ensure you have included details on (1) methods of sacrifice, (2) methods of anesthesia and/or analgesia, and (3) efforts to alleviate suffering.

3. We note that Figure 2 in your submission contain [map/satellite] images which may be copyrighted. All PLOS content is published under the Creative Commons Attribution License (CC BY 4.0), which means that the manuscript, images, and Supporting Information files will be freely available online, and any third party is permitted to access, download, copy, distribute, and use these materials in any way, even commercially, with proper attribution. For these reasons, we cannot publish previously copyrighted maps or satellite images created using proprietary data, such as Google software (Google Maps, Street View, and Earth). For more information, see our copyright guidelines: http://journals.plos.org/plosone/s/licenses-and-copyright.

We require you to either (1) present written permission from the copyright holder to publish these figures specifically under the CC BY 4.0 license, or (2) remove the figures from your submission:

1. You may seek permission from the original copyright holder of Figure 2 to publish the content specifically under the CC BY 4.0 license.

We recommend that you contact the original copyright holder with the Content Permission Form (http://journals.plos.org/plosone/s/file?id=7c09/content-permission-form.pdf) and the following text:

“I request permission for the open-access journal PLOS ONE to publish XXX under the Creative Commons Attribution License (CCAL) CC BY 4.0 (http://creativecommons.org/licenses/by/4.0/). Please be aware that this license allows unrestricted use and distribution, even commercially, by third parties. Please reply and provide explicit written permission to publish XXX under a CC BY license and complete the attached form.”

Please upload the completed Content Permission Form or other proof of granted permissions as an "Other" file with your submission.

In the figure caption of the copyrighted figure, please include the following text: “Reprinted from [ref] under a CC BY license, with permission from [name of publisher], original copyright [original copyright year].”

2. If you are unable to obtain permission from the original copyright holder to publish these figures under the CC BY 4.0 license or if the copyright holder’s requirements are incompatible with the CC BY 4.0 license, please either i) remove the figure or ii) supply a replacement figure that complies with the CC BY 4.0 license. Please check copyright information on all replacement figures and update the figure caption with source information. If applicable, please specify in the figure caption text when a figure is similar but not identical to the original image and is therefore for illustrative purposes only.

The following resources for replacing copyrighted map figures may be helpful:

USGS National Map Viewer (public domain): http://viewer.nationalmap.gov/viewer/

The Gateway to Astronaut Photography of Earth (public domain): http://eol.jsc.nasa.gov/sseop/clickmap/

Maps at the CIA (public domain): https://www.cia.gov/library/publications/the-world-factbook/index.html and https://www.cia.gov/library/publications/cia-maps-publications/index.html

NASA Earth Observatory (public domain): http://earthobservatory.nasa.gov/

Landsat: http://landsat.visibleearth.nasa.gov/

USGS EROS (Earth Resources Observatory and Science (EROS) Center) (public domain): http://eros.usgs.gov/#

Natural Earth (public domain): http://www.naturalearthdata.com/

If the reviewer comments include a recommendation to cite specific previously published works, please review and evaluate these publications to determine whether they are relevant and should be cited. There is no requirement to cite these works unless the editor has indicated otherwise.

[Note: HTML markup is below. Please do not edit.]

Reviewers' comments:

Reviewer's Responses to Questions

**Comments to the Author**

1. Is the manuscript technically sound, and do the data support the conclusions?

The manuscript must describe a technically sound piece of scientific research with data that supports the conclusions. Experiments must have been conducted rigorously, with appropriate controls, replication, and sample sizes. The conclusions must be drawn appropriately based on the data presented.

Reviewer #1: Partly

Reviewer #2: Partly

**********

2. Has the statistical analysis been performed appropriately and rigorously?

Reviewer #1: No

Reviewer #2: N/A

**********

3. Have the authors made all data underlying the findings in their manuscript fully available?

The PLOS Data policy requires authors to make all data underlying the findings described in their manuscript fully available without restriction, with rare exception (please refer to the Data Availability Statement in the manuscript PDF file). The data should be provided as part of the manuscript or its supporting information, or deposited to a public repository. For example, in addition to summary statistics, the data points behind means, medians and variance measures should be available. If there are restrictions on publicly sharing data—e.g. participant privacy or use of data from a third party—those must be specified.

Reviewer #1: Yes

Reviewer #2: Yes

**********

4. Is the manuscript presented in an intelligible fashion and written in standard English?

PLOS ONE does not copyedit accepted manuscripts, so the language in submitted articles must be clear, correct, and unambiguous. Any typographical or grammatical errors should be corrected at revision, so please note any specific errors here.

Reviewer #1: Yes

Reviewer #2: Yes

**********

5. Review Comments to the Author

Please use the space provided to explain your answers to the questions above. You may also include additional comments for the author, including concerns about dual publication, research ethics, or publication ethics. (Please upload your review as an attachment if it exceeds 20,000 characters)

Reviewer #1: The manuscript “Serological Evidence of SARS-CoV-2 Exposure in Marine Mammals in the United States between 2020 and 2025” is an interesting manuscript that reports evidence of SARS-CoV-2 exposure in marine mammals. The information presented by the authors is interesting and important to understand the ecology of SARS-CoV-2 and is clear that was a really big endeavor to achieve. However, despite the importance of this report, there are many technical issues that must be solved before being considered to be published. Also lab results interpretation is limited and as a consequence discussion is limited and for moments contradictory.

The main methodology issue is the performance of the ELISA. Authors do not describe in detail how this was performed. The reference that they present to validate the use of this test is for human test, not for a panmammalian as is claimed in the manuscript.

To properly evaluate the results detailed methodology is needed to detect possible pitfalls in the laboratory analyses. One of the most important details is the secondary antibody or protein used, as well as, all the controls used.

About the interpretation of the lab results, it is necessary to declare the limitation of the test used. For example, how specific are the primers used for the rt-pcr? If the primers are so specific and the strain is little different, the rt-pcr can lose the diagnostic, otherwise, a more degenerated primer can detect even different variants of the virus, making the result much more trustful.

Some data of the animals is missing. This is especially important for Zalophus, where the animals tested positive where actively captured. The site of capture is vital to understand how the animals get in contact with the virus.

Discussion should be rewrite and reinforce, in its actual form is just a superficial comparison. One important issue to be discussed is the lab results (based on the methodology used) and its limitations. The discussion is lack of analysis of the geographic distribution, difference of the species, antibody titer, among others.

Here some specific comments that could help to improve the manuscript:

Line 84-85: this sentence does not have enough sustain. The similitude does not explain why could be potentially infected at "low viral concentration", is it because it is similar to humans and humans can infect a low viral concentration?

Line 124: viral transport mediums need specification to know how long the virus or at least the virus RNA can last.

Line 135: declare the primers used and the range of detection for SARS-CoV-2. How specific are the primers just a specific strain of any SARS-CoV-2 strain.

Line 143: the 23 reference does not include a generic protocol for mammals, this article is about tests for humans.

Line 144-145: Authors omitted the most important part of the protocol, since the previous reference is for testing in humans.

Clearly and highly detailed protocol must be included, with emphasis in secondary antibody. The secondary antibody (or protein) must be capable of recognizing FC from immunoglobulins of mammals. This part is crucial to understand the effectiveness of the serological test.

If there is not a previous use of this methodology, authors must include the standardization phase as a supplementary material.

Line 147: what the authors used has negative controls?

Line 149: What does “healthy” mean for the authors? How could the authors rule-out a subclinical or previous exposition?

Line 149: why the authors selected this species and no others?

Line 156: If a validation (or more precisely comparison) is attempted, a statistical test must be run, such as a concordance test such as kappa. And in this way, positive and negative samples must be included, not just positive.

Line 163: Controls for the neutralization test must be declared. How the authors can test the true inactivation of the serum. Some carnivore serums are still killing cells after heat (56oC) inactivation.

Line 165: a data analyses section is necessary

Line 172: authors can not ensure if the virus is active just by pcr. But you can say that at least there is some RNA of the virus in the sample.

Line 174: even when authors already mention how many samples analyzed, it would be helpful to put 18 out of how many, and of course a prevalence or frequency along with confidence intervals.

Line 175: since CA sea lions come from active capture, it is important to declare and to discuss where the animals were captured. Especially since this species is more likely to have a natural infection. Is this site close to human settlements?

Line 178: Same 12 samples of the confirmatory ELISA?

Line 180: the first sentence is confusing, it looks like authors test both condition stranded and active capture. However, just samples from grey came from both sources, active and stranded, so this declaration implies that you evaluate both.

Line 187: this raises some questions. Samples were analyzed separately? This is increasing the number of positive samples. Authors must clean the data base specially of the resampling individuals where there is not so much time difference.

Line 191: is not 3/7 since one was not tested, must be 3/6

Line 195-197: some of the table legend was already in the methodology, not necessary here.

Line 199: is necessary to include confidence interval

Line 201: Is important to mention that you are considering a positive animal (or sample) when it was positive to ELISA and SN.

Line 202-205: Authors have data to compare prevalence between species, maybe sex and age, why the authors did not? A simple x-square test can solve this. It was a big effort to lose this information.

Line 229: the specific clinical history of the positive animals, specially from those stranded, are really important to evaluate the odds of get infected in the rehabilitation center. Antibodies usually need at least 21 to get formed, in this way if the sample was get before that time, seroconversion should be before the contact to humans.

Line 250-252: Make clear what is the relevance of this for a SARS-CoV-2 study, particularly the final part of this paragraph “the clinical significance of the HSCoV remains unclear due to incomplete sampling during the outbreak”

Line 255-257: No agree with this, new variants are more specific to humans, original ones had more potential to cross transition.

Line 274: “using contemporary SARS-CoV-2 strains in the VNT could have increased its sensitivity” indeed but also if the antibodies of your study came from cross reaction, new strain could detect even lower prevalence.

Figures: quality must be improved.

I recommend to the authors to explore to the natural dynamics of immunoglobulin production and decay. Also, difference between neutralizing antibodies and those antibodies that have not neutralizing activity. To considered exposure to the virus, this difference is important. In this way, almost all antibodies need exposure to the antigen to be produce, but not necessary all produce neutralizing activity (i.e. protection to the pathogen).

It is important to know if your secondary antibody allows you to identify the isotype of immunoglobulin that you found in the ELISA test. Is not the same interpretation if you find IgG or IgM.

Finally, the description of other potential coronaviruses present in marine mammals is important and even more, how probable is the cross reaction with SARS-CoV-2. This is a key point since there are no other complementary techniques.

Reviewer #2: Overall, this article is well written and presents interesting findings. However, several methodological and interpretive points require clarification to strengthen confidence in the results.

General Comments

# The authors do not mention whether any pan coronavirus or consensus coronavirus PCR assays were performed on the swabs. Given the possibility of cross reactivity in serological assays, this information is important for interpreting the results.

# Because serological assays can yield false positives, the study design and conclusions should be interpreted cautiously. The authors should report the sensitivity of the ELISA RBD assay particularly in any relevant mammalian species to help readers assess the likelihood of missed positives.

# Virus neutralization testing (VNT) was not performed on all serum samples. The authors should clarify how they calculated the percentage of neutralizing antibodies and whether these analyses are appropriate given that VNT was only conducted on indirect ELISA RBD–positive samples.

# Including a larger number of pre pandemic serum samples would strengthen the study by helping determine whether the assays produce any unexpected positives and by increasing confidence in the VNT results.

# The absence of evidence for active infection makes it difficult to interpret the low to moderate VNT titers observed in a few animals. Additionally, different VNT formats can vary in false positive and false negative rates, which should be acknowledged.

Line Specific Comments

Lines 142–144: What is the sensitivity of the ELISA RBD assay, particularly for any relevant mammalian species? Including this information would help readers assess the likelihood of false negatives. If sensitivity is low, some positive animals may have been missed; if high, it strengthens confidence in the findings.

Lines 149–150: Only one pre pandemic serum sample was used as a negative control. Is it possible to include additional pre pandemic samples? This would help determine whether any of the assays produce unexpected positives and would strengthen the overall conclusions.

Line 156: If I understand correctly, all serum samples were first screened using the ELISA RBD assay, and only RBD positive samples were subsequently tested by ELISA Spike and VNT. If this is accurate, please clarify the reference to “20 positive samples (n = 20)” here. In the Results section, you report 18 ELISA RBD positive samples. Reconciling these numbers would improve clarity.

Lines 166–168: Please specify in the results section the number of animals tested by PCR and by ELISA. Although the table provides totals, including these figures directly in the text would improve readability. For example, briefly state the total number of animals tested, how many underwent PCR, how many were tested serologically, and how many received both.

Lines 198–201: As I understand it, VNT was performed only on ELISA-RBD positive samples. If so, analyses comparing VNT results across ELISA RBD negative animals may not be appropriate unless VNT was performed on all samples or the ELISA RBD assay is known to be 100% sensitive.

Lines 204–205: The same concern applies here: analyses involving VNT results should account for the fact that VNT was not conducted on ELISA RBD negative samples.

**********

6. PLOS authors have the option to publish the peer review history of their article (what does this mean?). If published, this will include your full peer review and any attached files.

If you choose “no”, your identity will remain anonymous but your review may still be made public.

**Do you want your identity to be public for this peer review?**  For information about this choice, including consent withdrawal, please see our Privacy Policy.

Reviewer #1: No

Reviewer #2: No

**********

[NOTE: If reviewer comments were submitted as an attachment file, they will be attached to this email and accessible via the submission site. Please log into your account, locate the manuscript record, and check for the action link "View Attachments". If this link does not appear, there are no attachment files.]

To ensure your figures meet our technical requirements, please review our figure guidelines: https://journals.plos.org/plosone/s/figures

You may also use PLOS’s free figure tool, NAAS, to help you prepare publication quality figures: https://journals.plos.org/plosone/s/figures#loc-tools-for-figure-preparation.

NAAS will assess whether your figures meet our technical requirements by comparing each figure against our figure specifications.

# Author comment: Author response to Decision Letter 1

**DOI:** 10.1371/journal.pone.0351734.r002

1 Apr 2026

Comments from Academic Editor

1. Please ensure that your manuscript meets PLOS ONE's style requirements, including those for file naming. The PLOS ONE style templates can be found at

https://journals.plos.org/plosone/s/file?id=wjVg/PLOSOne_formatting_sample_main_body.pdf and

https://journals.plos.org/plosone/s/file?id=ba62/PLOSOne_formatting_sample_title_authors_affiliations.pdf

Response: We have revised the manuscript to ensure that it complies with the style requirements of PLOS ONE.

2. To comply with PLOS One submissions requirements, in your Methods section, please provide additional information regarding the experiments involving animals and ensure you have included details on (1) methods of sacrifice, (2) methods of anesthesia and/or analgesia, and (3) efforts to alleviate suffering.

Response: Thank you for this comment.

No animals were sacrificed or experimentally manipulated for the purposes of this study. Samples were obtained opportunistically from animals admitted to rehabilitation centers, from stranded animals found dead, and from animals captured and released as part of monitoring programs, during which biological samples were collected. We did not perform any procedures involving anesthesia or euthanasia. All animal handling was performed by trained marine mammal rehabilitation personnel following standard animal welfare protocols. This information has now been clarified in the Methods section on page 6, lines 129 - 131.

3. We note that Figure 2 in your submission contain [map/satellite] images which may be copyrighted. All PLOS content is published under the Creative Commons Attribution License (CC BY 4.0), which means that the manuscript, images, and Supporting Information files will be freely available online, and any third party is permitted to access, download, copy, distribute, and use these materials in any way, even commercially, with proper attribution. For these reasons, we cannot publish previously copyrighted maps or satellite images created using proprietary data, such as Google software (Google Maps, Street View, and Earth). For more information, see our copyright guidelines: http://journals.plos.org/plosone/s/licenses-and-copyright.

Response: Thank you for highlighting this point. The base map used in Figure 2 was obtained from the U.S. Census Bureau (https://www.census.gov/) and is in the public domain. We have now added the following statement to the Figure 2 caption (page 12, lines 255 – 256): “State boundaries were derived from public domain U.S. Census Bureau TIGER/Line shapefiles”. The corresponding citation has also been included in the reference list (Reference [26]).

If the reviewer comments include a recommendation to cite specific previously published works, please review and evaluate these publications to determine whether they are relevant and should be cited. There is no requirement to cite these works unless the editor has indicated otherwise

Comments from Reviewer #1

Reviewer #1: The manuscript “Serological Evidence of SARS-CoV-2 Exposure in Marine Mammals in the United States between 2020 and 2025” is an interesting manuscript that reports evidence of SARS-CoV-2 exposure in marine mammals. The information presented by the authors is interesting and important to understand the ecology of SARS-CoV-2 and is clear that was a really big endeavor to achieve. However, despite the importance of this report, there are many technical issues that must be solved before being considered to be published. Also lab results interpretation is limited and as a consequence discussion is limited and for moments contradictory.

The main methodology issue is the performance of the ELISA. Authors do not describe in detail how this was performed. The reference that they present to validate the use of this test is for human test, not for a panmammalian as is claimed in the manuscript.

To properly evaluate the results detailed methodology is needed to detect possible pitfalls in the laboratory analyses. One of the most important details is the secondary antibody or protein used, as well as, all the controls used.

Response: Thank you for this comment. We have expanded the ELISA methodology in the revised manuscript to provide additional experimental details and clarify the controls used. Although the SARS-CoV-2 ELISA was initially developed to detect antibodies against the receptor-binding domain (RBD) of SARS-CoV-2 in human sera (ref: https://doi.org/10.1002/cpmc.100), the assay was previously adapted and optimized in our laboratory for the detection of SARS-CoV-2 antibodies in ferret samples (ref: https://doi.org/10.1073/pnas.2025601118). Before this study, the ELISA assay was also validated for detecting IgG antibodies in serum samples from multiple mammalian orders, including marine mammals. These validation data are now provided in Supplementary Figure 1 and are referenced in the Methods section (page 8, lines 159 – 160).

To enable antibody detection across mammalian species, we used Pierce Recombinant Protein A/G Peroxidase Conjugate as the secondary antibody, and this information was added to the Methods section (page 8, line 168). This fusion protein combines the IgG-binding domains of Protein A and Protein G and binds the Fc region of IgG antibodies from a broad range of mammalian species, making it suitable for multi-species serological studies.

As a positive control, we used serum from spike (S) protein-immunized alpacas, as marine mammal sera with confirmed SARS-CoV-2 antibodies were not available. As negative controls, we used pre-pandemic gray seal sera collected during avian influenza monitoring programs in 2018. This information is provided in the Methods section (page 8, lines 174-175).

About the interpretation of the lab results, it is necessary to declare the limitation of the test used. For example, how specific are the primers used for the rt-pcr? If the primers are so specific and the strain is little different, the rt-pcr can lose the diagnostic, otherwise, a more degenerated primer can detect even different variants of the virus, making the result much more trustful.

Response: Thank you for this comment. The RT-PCR assay used in this study targeted the ORF1b (NSP14) region of SARS-CoV-2, which is highly conserved among SARS-CoV-2 lineages and has been widely used for sensitive detection of the virus. In addition, β-actin was amplified as an internal control to confirm RNA integrity and sample quality.

We acknowledge that the sensitivity of RT-PCR assays relies on primer binding to conserved genomic regions, and mutations in SARS-CoV-2 primer target sites can reduce assay sensitivity and potentially result in false negatives. However, the ORF1b region (encoding non-structural protein 14) targeted in our assay is among the most conserved regions of the SARS-CoV-2 genome, which reduces the likelihood that sequence variation substantially affected detection sensitivity (https://pmc.ncbi.nlm.nih.gov/articles/PMC9933857/). This limitation has now been included in the Discussion section on page 15, lines 326-330.

Some data of the animals is missing. This is especially important for Zalophus, where the animals tested positive where actively captured. The site of capture is vital to understand how the animals get in contact with the virus.

Response: In this study, we collaborated with more than 22 marine mammal rehabilitation centers as well as personnel from the National Oceanic and Atmospheric Administration (NOAA). Because samples were collected through multiple agencies and under varying field conditions and onsite protocols, metadata associated with individual animals varies across sample sources. However, to address the reviewer’s concern, we have added information in the Discussion section describing the geographic context in which California sea lions were sampled, which may help interpret potential exposure to the virus. The following sentence has been added to the Discussion section as follows (page 14, Lines 306-309): Moreover, the California sea lions sampled in this study were captured and released in San Miguel Island, where they may interact with humans through activities such as ecotourism and research operations. These human-animal interfaces may increase opportunities for exposure to SARS-CoV-2 through spillover from infected individuals.

Discussion should be rewrite and reinforce, in its actual form is just a superficial comparison. One important issue to be discussed is the lab results (based on the methodology used) and its limitations. The discussion is lack of analysis of the geographic distribution, difference of the species, antibody titer, among others.

Response: Thank you for this valuable comment. We have substantially revised and expanded the Discussion to strengthen the interpretation of our findings. Specifically, we now provide a more detailed analysis of the serological results in the context of the methodology used, including the strengths and limitations of the ELISA and virus neutralization assays (page 15, lines 326-351). We also expanded the discussion to better examine patterns in the data, including geographic distribution of seropositive animals, differences among species, and variation in antibody titers. In addition, we incorporated relevant examples from rehabilitation histories and ecological contexts that may help explain potential exposure to SARS-CoV-2 (page 14, lines 301-320).

Here some specific comments that could help to improve the manuscript:

Line 84-85: this sentence does not have enough sustain. The similitude does not explain why could be potentially infected at "low viral concentration", is it because it is similar to humans and humans can infect a low viral concentration?

Response: Marine mammal species were predicted to be susceptible to SARS-CoV-2 based on in silico modeling of the binding affinity between the viral spike protein and the cellular receptor ACE2, using human ACE2 as a reference. We intended to emphasize that certain marine mammal species may be highly susceptible to SARS-CoV-2 infection based on those studies. However, we agree with the reviewer that the original sentence could lead to confusion. To improve clarity, we removed the sentence “the similarity level of their ACE2 receptor with human ACE2, indicating SARS-CoV-2 infection could potentially be caused by a low viral concentration. The revised text now reads as follows (page 4, lines 85–87): Numerous marine mammal species, including most pinniped and cetacean species and particularly harbor seals, were predicted to be highly susceptible to SARS-CoV-2 infection based on in silico modeling of SARS-CoV-2 spike protein binding affinity to their ACE2 receptors.

Line 124: viral transport mediums need specification to know how long the virus or at least the virus RNA can last.

Response: The composition of the viral transport medium has been specified in the Methods section (page 6, line 134-135).

Line 135: declare the primers used and the range of detection for SARS-CoV-2. How specific are the primers just a specific strain of any SARS-CoV-2 strain.

Response: All samples were tested for SARS-CoV-2 viral RNA using a real-time RT-qPCR assay targeting the open reading frame 1b non-structural protein 14 (ORF1b-nsp14) region, using the HKU-ORF1 primer–probe set described by Chu et al. [23] (Forward 5′-TGGGGYTTTACRGGTAACCT-3′, Reverse 5′-AACRCGCTTAACAAAGCACTC-3′, Probe 5′-FAM-TAGTTGTGATGCWATCATGACTAG-TAMRA-3′). This assay targets a highly conserved region of the SARS-CoV-2 genome and is designed to detect diverse SARS-CoV-2 lineages rather than a specific strain. We have specified the primer–probe set used for SARS-CoV-2 detection in the Methods section (page 7, lines 146-148).

Line 143: the 23 reference does not include a generic protocol for mammals, this article is about tests for humans.

Response: As noted above, the ELISA protocol implemented in this study was previously applied by K. Sawatzki et al. (https://www.pnas.org/doi/10.1073/pnas.2025601118) to detect SARS-CoV-2 antibodies in ferrets, demonstrating its applicability to non-human mammalian samples. Prior to this study, the assay was validated in-house to detect IgG from a wide range of mammals, including marine mammals. To address the reviewer’s concern and improve clarity and reproducibility, we have expanded the methodological description of the ELISA protocol in the Methods section (page 8, lines 159 – 160) and added Supporting Information Figure S1 to illustrate IgG detection across several species.

Line 144-145: Authors omitted the most important part of the protocol, since the previous reference is for testing in humans.

Response: The ELISA protocol has been expanded to include additional methodological details (page 8, lines 159-170)

Clearly and highly detailed protocol must be included, with emphasis in secondary antibody. The secondary antibody (or protein) must be capable of recognizing FC from immunoglobulins of mammals. This part is crucial to understand the effectiveness of the serological test.

Response: Information regarding the secondary antibody, specifically the Pierce Recombinant Protein A/G Peroxidase Conjugate, has now been included in the Methods section (page 8, line 168).

If there is not a previous use of this methodology, authors must include the standardization phase as a supplementary material.

Response: The ELISA protocol implemented in this study was previously adapted and used by K. Sawatzki et al. (https://www.pnas.org/doi/10.1073/pnas.2025601118) to detect SARS-CoV-2 antibodies in ferrets. However, as noted above, the ELISA assay was validated in-house for detecting IgG antibodies in serum samples from several wildlife mammalian species. The validation data are shown in Supplementary Figure 1 and referenced in the Methods section (page 8, lines 159 – 160).

Line 147: what the authors used has negative controls?

Response: Pre-pandemic serum samples collected from gray seals in 2018 during influenza monitoring programs were used as negative controls, allowing us to establish the background signal and support the specificity of the ELISA assay. This information is provided in the Methods section (page 8, line 175).

Line 149: What does “healthy” mean for the authors? How could the authors rule-out a subclinical or previous exposition?

Response: We agree with the reviewer that subclinical exposure cannot be completely ruled out. We intended to indicate that these serum samples were collected before the emergence of SARS-CoV-2, making prior exposure to this virus highly unlikely. To avoid ambiguity, the term “healthy” (page 8, line 175) has been removed from the manuscript.

Line 149: why the authors selected this species and no others?

Response: The positive control reagent was kindly provided by our colleague on the Tufts campus, Dr. Charles Shoemaker. Unrelated to the work reported here, Dr. Shoemaker has undertaken studies to develop camelid antibodies against SARS-CoV-2, wherein alpacas were immunized with the spike (S) protein and developed robust antibody responses to the S protein.

Line 156: If a validation (or more precisely comparison) is attempted, a statistical test must be run, such as a concordance test such as kappa. And in this way, positive and negative samples must be included, not just positive.

Response: Thank you for this comment. We agree that statistical concordance analyses, such as Cohen’s kappa, are appropriate for formally comparing the diagnostic performance of two assays and require both positive and negative samples. In the present study, however, the neutralization assay was not used in comparison to the ELISA, but rather to confirm the presence of functional neutralizing antibodies in ELISA-positive samples. Therefore, only ELISA-positive sera were selected for neutralization testing. Because ELISA-negative samples were not included in this analysis, a concordance test, such as kappa, could not be performed.

Line 163: Controls for the neutralization test must be declared. How the authors can test the true inactivation of the serum. Some carnivore serums are still killing cells after heat (56oC) inactivation.

Response: Each plate included a virus-only control, a cell-only contro

Attachment Submitted filename: Response to Reviewers.pdf

# Peer review: Decision Letter 1

**DOI:** 10.1371/journal.pone.0351734.r003

**Authors:** Stephen Raverty, Stephen Raverty

4 May 2026

PONE-D-26-02880R1

Serological Evidence of SARS-CoV-2 Exposure in Marine Mammals in the United States between 2020 and 2025

PLOS One

Dear Dr. Sanogo,

Thank you for submitting your manuscript to PLOS ONE. After careful consideration, we feel that it has merit but does not fully meet PLOS ONE’s publication criteria as it currently stands. Therefore, we invite you to submit a revised version of the manuscript that addresses the points raised during the review process.

Please submit your revised manuscript by Jun 18 2026 11:59PM. If you will need more time than this to complete your revisions, please reply to this message or contact the journal office at plosone@plos.org. When you're ready to submit your revision, log on to https://www.editorialmanager.com/pone/ and select the 'Submissions Needing Revision' folder to locate your manuscript file.

Please include the following items when submitting your revised manuscript:

- A letter that responds to each point raised by the academic editor and reviewer(s). You should upload this letter as a separate file labeled 'Response to Reviewers'.
- A marked-up copy of your manuscript that highlights changes made to the original version. You should upload this as a separate file labeled 'Revised Manuscript with Track Changes'.
- An unmarked version of your revised paper without tracked changes. You should upload this as a separate file labeled 'Manuscript'.

If you would like to make changes to your financial disclosure, please include your updated statement in your cover letter. Guidelines for resubmitting your figure files are available below the reviewer comments at the end of this letter.

If applicable, we recommend that you deposit your laboratory protocols in protocols.io to enhance the reproducibility of your results. Protocols.io assigns your protocol its own identifier (DOI) so that it can be cited independently in the future. For instructions see: https://journals.plos.org/plosone/s/submission-guidelines#loc-laboratory-protocols. Additionally, PLOS ONE offers an option for publishing peer-reviewed Lab Protocol articles, which describe protocols hosted on protocols.io. Read more information on sharing protocols at https://plos.org/protocols?utm_medium=editorial-email&utm_source=authorletters&utm_campaign=protocols.

As the corresponding author, your ORCID iD is verified in the submission system and will appear in the published article. PLOS supports the use of ORCID, and we encourage all coauthors to register for an ORCID iD and use it as well. Please encourage your coauthors to verify their ORCID iD within the submission system before final acceptance, as unverified ORCID iDs will not appear in the published article. *Only*  the individual author can complete the verification step; PLOS staff *cannot*  verify ORCID iDs on behalf of authors.

We look forward to receiving your revised manuscript.

Kind regards,

Stephen Raverty

Academic Editor

PLOS One

Journal Requirements:

If the reviewer comments include a recommendation to cite specific previously published works, please review and evaluate these publications to determine whether they are relevant and should be cited. There is no requirement to cite these works unless the editor has indicated otherwise.

Please review your reference list to ensure that it is complete and correct. If you have cited papers that have been retracted, please include the rationale for doing so in the manuscript text, or remove these references and replace them with relevant current references. Any changes to the reference list should be mentioned in the rebuttal letter that accompanies your revised manuscript. If you need to cite a retracted article, indicate the article’s retracted status in the References list and also include a citation and full reference for the retraction notice.

**Additional Editor Comments:**

There are minor edits which should addressed prior to publication of this manuscript. Thank you for your detailed responses to the reviewers comments.

[Note: HTML markup is below. Please do not edit.]

Reviewers' comments:

Reviewer's Responses to Questions

**Comments to the Author**

1. If the authors have adequately addressed your comments raised in a previous round of review and you feel that this manuscript is now acceptable for publication, you may indicate that here to bypass the “Comments to the Author” section, enter your conflict of interest statement in the “Confidential to Editor” section, and submit your "Accept" recommendation.

Reviewer #1: All comments have been addressed

Reviewer #2: All comments have been addressed

**********

2. Is the manuscript technically sound, and do the data support the conclusions?

The manuscript must describe a technically sound piece of scientific research with data that supports the conclusions. Experiments must have been conducted rigorously, with appropriate controls, replication, and sample sizes. The conclusions must be drawn appropriately based on the data presented.

Reviewer #1: Yes

Reviewer #2: Yes

**********

3. Has the statistical analysis been performed appropriately and rigorously?

Reviewer #1: Yes

Reviewer #2: Yes

**********

4. Have the authors made all data underlying the findings in their manuscript fully available?

The PLOS Data policy requires authors to make all data underlying the findings described in their manuscript fully available without restriction, with rare exception (please refer to the Data Availability Statement in the manuscript PDF file). The data should be provided as part of the manuscript or its supporting information, or deposited to a public repository. For example, in addition to summary statistics, the data points behind means, medians and variance measures should be available. If there are restrictions on publicly sharing data—e.g. participant privacy or use of data from a third party—those must be specified.

Reviewer #1: Yes

Reviewer #2: Yes

**********

5. Is the manuscript presented in an intelligible fashion and written in standard English?

PLOS ONE does not copyedit accepted manuscripts, so the language in submitted articles must be clear, correct, and unambiguous. Any typographical or grammatical errors should be corrected at revision, so please note any specific errors here.

Reviewer #1: Yes

Reviewer #2: Yes

**********

6. Review Comments to the Author

Please use the space provided to explain your answers to the questions above. You may also include additional comments for the author, including concerns about dual publication, research ethics, or publication ethics. (Please upload your review as an attachment if it exceeds 20,000 characters)

Reviewer #1: Authors solve all queries. Just a few minor comments.

It will be desirable to include representative figures of the controls, positives and negatives samples of the seroneutralization test.

Protein A/G binds to the FC of any immunoglobulin, is not specific for IgG, therefore authors are reporting total Ig.

Inganäs, M. (1981). Comparison of Mechanisms of Interaction between Protein A from Staphylococcus aureus and Human Monoclonal IgG, IgA and IgM in Relation to the Classical Fcγ and the Alternative F (ab') 2γ Protein A Interactions. Scandinavian journal of immunology, 13(4), 343-352.

Scientific names should be used more than common names, once both names were mentioned.

Reviewer #2: (No Response)

**********

7. PLOS authors have the option to publish the peer review history of their article (what does this mean?). If published, this will include your full peer review and any attached files.

If you choose “no”, your identity will remain anonymous but your review may still be made public.

**Do you want your identity to be public for this peer review?**  For information about this choice, including consent withdrawal, please see our Privacy Policy.

Reviewer #1: No

Reviewer #2: No

**********

[NOTE: If reviewer comments were submitted as an attachment file, they will be attached to this email and accessible via the submission site. Please log into your account, locate the manuscript record, and check for the action link "View Attachments". If this link does not appear, there are no attachment files.]

To ensure your figures meet our technical requirements, please review our figure guidelines: https://journals.plos.org/plosone/s/figures

You may also use PLOS’s free figure tool, NAAS, to help you prepare publication quality figures: https://journals.plos.org/plosone/s/figures#loc-tools-for-figure-preparation.

NAAS will assess whether your figures meet our technical requirements by comparing each figure against our figure specifications.

# Author comment: Author response to Decision Letter 2

**DOI:** 10.1371/journal.pone.0351734.r004

7 May 2026

Comments from Academic Editor

There are minor edits which should addressed prior to publication of this manuscript. Thank you for your detailed responses to the reviewers' comments.

Response: We have addressed the reviewers’ comments below.

Comments from Reviewer #1

Reviewer #1: Authors solve all queries. Just a few minor comments.

Response: Thank you!

It will be desirable to include representative figures of the controls, positives and negatives samples of the seroneutralization test.

Response: We thank the reviewer for this helpful suggestion and agree that inclusion of representative images would enhance the presentation of the assay. However, representative images from the seroneutralization (SN) assay were not systematically recorded at the time of the experiments and therefore cannot be provided retrospectively. To nevertheless provide a visual representation of the samples’ serological profiles, we have included an image of the corresponding ELISA plate in the Supplementary Material (Fig. S2). For reference, we also provide in the Response to Reviewers file a representative image of the SN assays in our lab.

In addition, SN results were independently evaluated by two operators, and this has now been explicitly stated in the Methods section (line 184) to strengthen confidence in the scoring procedure.

Protein A/G binds to the FC of any immunoglobulin, is not specific for IgG, therefore authors are reporting total Ig.

Inganäs, M. (1981). Comparison of Mechanisms of Interaction between Protein A from Staphylococcus aureus and Human Monoclonal IgG, IgA and IgM in Relation to the Classical Fcγ and the Alternative F (ab') 2γ Protein A Interactions. Scandinavian journal of immunology, 13(4), 343-352.

Response:

We agree with the reviewer that Protein A/G may bind multiple immunoglobulin classes and is not strictly IgG-specific. In response, we have revised the manuscript at line 154 to clarify that our assay detects total immunoglobulins rather than IgG specifically.

Scientific names should be used more than common names, once both names were mentioned.

Response: We thank the reviewer for this suggestion and agree that consistent use of scientific names improves clarity and precision. We have revised the manuscript to preferentially use scientific names after their first mention alongside common names.

Comments from Reviewer #2

All comments have been addressed.

Response: Thank you!

Attachment Submitted filename: Response to Reviewers_R2.pdf

# Peer review: Decision Letter 2

**DOI:** 10.1371/journal.pone.0351734.r005

**Authors:** Stephen Raverty, Stephen Raverty, Mohamed Abousenna

2 Jun 2026

Serological Evidence of SARS-CoV-2 Exposure in Marine Mammals in the United States between 2020 and 2025

PONE-D-26-02880R2

Dear Dr. Sanogo,

We’re pleased to inform you that your manuscript has been judged scientifically suitable for publication and will be formally accepted for publication once it meets all outstanding technical requirements.

Within one week, you’ll receive an e-mail detailing the required amendments. When these have been addressed, you’ll receive a formal acceptance letter and your manuscript will be scheduled for publication.

An invoice will be generated when your article is formally accepted. Please note, if your institution has a publishing partnership with PLOS and your article meets the relevant criteria, all or part of your publication costs will be covered. Please make sure your user information is up-to-date by logging into Editorial Manager at Editorial Manager® and clicking the ‘Update My Information' link at the top of the page. For questions related to billing, please contact billing support.

If your institution or institutions have a press office, please notify them about your upcoming paper to help maximize its impact. If they’ll be preparing press materials, please inform our press team as soon as possible -- no later than 48 hours after receiving the formal acceptance. Your manuscript will remain under strict press embargo until 2 pm Eastern Time on the date of publication. For more information, please contact onepress@plos.org.

Kind regards,

Mohamed Samy Abousenna, Ph.D

Academic Editor

PLOS One

Additional Editor Comments (optional):

Reviewers' comments:

Reviewer's Responses to Questions

**Comments to the Author**

1. If the authors have adequately addressed your comments raised in a previous round of review and you feel that this manuscript is now acceptable for publication, you may indicate that here to bypass the “Comments to the Author” section, enter your conflict of interest statement in the “Confidential to Editor” section, and submit your "Accept" recommendation.

Reviewer #1: All comments have been addressed

Reviewer #2: All comments have been addressed

Reviewer #3: All comments have been addressed

Reviewer #4: All comments have been addressed

**********

2. Is the manuscript technically sound, and do the data support the conclusions?

The manuscript must describe a technically sound piece of scientific research with data that supports the conclusions. Experiments must have been conducted rigorously, with appropriate controls, replication, and sample sizes. The conclusions must be drawn appropriately based on the data presented.

Reviewer #1: Yes

Reviewer #2: Yes

Reviewer #3: Yes

Reviewer #4: Yes

**********

3. Has the statistical analysis been performed appropriately and rigorously?

Reviewer #1: Yes

Reviewer #2: Yes

Reviewer #3: Yes

Reviewer #4: Yes

**********

4. Have the authors made all data underlying the findings in their manuscript fully available?

The PLOS Data policy requires authors to make all data underlying the findings described in their manuscript fully available without restriction, with rare exception (please refer to the Data Availability Statement in the manuscript PDF file). The data should be provided as part of the manuscript or its supporting information, or deposited to a public repository. For example, in addition to summary statistics, the data points behind means, medians and variance measures should be available. If there are restrictions on publicly sharing data—e.g. participant privacy or use of data from a third party—those must be specified.

Reviewer #1: Yes

Reviewer #2: Yes

Reviewer #3: Yes

Reviewer #4: Yes

**********

5. Is the manuscript presented in an intelligible fashion and written in standard English?

PLOS ONE does not copyedit accepted manuscripts, so the language in submitted articles must be clear, correct, and unambiguous. Any typographical or grammatical errors should be corrected at revision, so please note any specific errors here.

Reviewer #1: Yes

Reviewer #2: Yes

Reviewer #3: Yes

Reviewer #4: Yes

**********

6. Review Comments to the Author

Please use the space provided to explain your answers to the questions above. You may also include additional comments for the author, including concerns about dual publication, research ethics, or publication ethics. (Please upload your review as an attachment if it exceeds 20,000 characters)

Reviewer #1: (No Response)

Reviewer #2: (No Response)

Reviewer #3: Overall, the revised manuscript is substantially improved, and most of the previous comments have been adequately addressed. I have one minor suggestion regarding the concluding paragraph. The discussion around the possible establishment of endemic infection and subsequent viral evolution in marine mammal hosts may imply a stronger conclusion than is directly supported by the current data, given the absence of PCR-positive animals and the limited serological evidence presented. Therefore, the authors may wish to consider slightly softening this wording so that the conclusions remain closely aligned with the finding

Reviewer #4: Reviewer comment #1

1. Abstract and Introduction

The Introduction is well written and provides a good background on SARS-CoV-2 spillover and marine mammal susceptibility. However, it does not fully justify the study design, hypothesis, significance of serology, or the interpretation of findings reported in the abstract as follows:

1.1. Aim and hypothesis: To make the study scientifically stronger, the author should sufficiently define the objectives and hypothesis and emphasize that this study is the first to provide serological evidence of SARS-CoV-2 exposure in marine mammals in the United States.

1.2. There is a lack of a clear rationale for combining PCR and serology. As the abstract reports both RT-qPCR and serological testing, the Introduction mainly discusses infection risk. It does not clearly explain why antibody surveillance is essential in addition to PCR testing.

1.3. The abstract emphasizes that this is the “first serological evidence” of SARS-CoV-2 exposure in marine mammals in the United States. However, the Introduction does not adequately explain why serological detection alone is epidemiologically important.

Could the authors explain the significance of detecting neutralizing antibodies in marine mammals despite the absence of active infection by RT-qPCR?

1.4. Additionally, given the gap, the authors should note which surveillance studies have already been conducted globally and which gaps remain.

Reviewer comment #2

2. Methodology

The methodology is strong and suitable for its objectives, with notable strengths in laboratory workflow and confirmatory testing. The study demonstrates excellent adherence to ethical standards. It includes a large, diverse sample and a rigorous serological testing strategy.

Below are some improvements that could be clarified to enhance the study's rigour:

2.1. Have the authors recorded any demographic data for the sampled animals, such as sex, age, and clinical condition, since environmental factors may influence serological findings? Please add demographic and environmental variables.

2.2. According to Table 1, the geographic and temporal distributions of the samples collected between 2020 and 2025 were not included in the table. Could the authors add additional information regarding the sampling locations and yearly distribution of the collected samples, if applicable?

Reviewer comment #3

3. Results

The results are clearly displayed and well structured. There are no major methodological errors.

Reviewer comment #4

4. Discussion

The discussion is well written and has an excellent start emphasizing that this study is the first in investigating SARS-CoV-2 in several marine mammal species on both the East and West coasts of the U.S.

Reviewer comment #5

5. Figures

Figures 1 and 2 need to be prepared in Illustrator to ensure the highest resolution. Alternatively, they can be exported in high-quality vector formats, such as PDF or EPS, which are preferred for publication.

**********

7. PLOS authors have the option to publish the peer review history of their article (what does this mean?). If published, this will include your full peer review and any attached files.

If you choose “no”, your identity will remain anonymous but your review may still be made public.

**Do you want your identity to be public for this peer review?**  For information about this choice, including consent withdrawal, please see our Privacy Policy.

Reviewer #1: No

Reviewer #2: No

Reviewer #3: No

Reviewer #4: **Yes:** Ruba Abdulrahman Ashy

**********

Attachment Submitted filename: Comments-PLOs One-PONE-D-26-02880_R2. Reviewer 2.pdf

# Editor report: Acceptance letter

**DOI:** 10.1371/journal.pone.0351734.r006

**Authors:** Stephen Raverty, Stephen Raverty, Mohamed Abousenna

PONE-D-26-02880R2

PLOS One

Dear Dr. Sanogo,

I'm pleased to inform you that your manuscript has been deemed suitable for publication in PLOS One. Congratulations! Your manuscript is now being handed over to our production team.

At this stage, our production department will prepare your paper for publication. This includes ensuring the following:

* All references, tables, and figures are properly cited

* All relevant supporting information is included in the manuscript submission,

* There are no issues that prevent the paper from being properly typeset

You will receive further instructions from the production team, including instructions on how to review your proof when it is ready. Please keep in mind that we are working through a large volume of accepted articles, so please give us a few days to review your paper and let you know the next and final steps.

Lastly, if your institution or institutions have a press office, please let them know about your upcoming paper now to help maximize its impact. If they'll be preparing press materials, please inform our press team within the next 48 hours. Your manuscript will remain under strict press embargo until 2 pm Eastern Time on the date of publication. For more information, please contact onepress@plos.org.

You will receive an invoice from PLOS for your publication fee after your manuscript has reached the completed accept phase. If you receive an email requesting payment before acceptance or for any other service, this may be a phishing scheme. Learn how to identify phishing emails and protect your accounts at https://explore.plos.org/phishing.

If we can help with anything else, please email us at customercare@plos.org.

Thank you for submitting your work to PLOS ONE and supporting open access.

Kind regards,

PLOS ONE Editorial Office Staff

on behalf of

Dr. Mohamed Samy Abousenna

Academic Editor

PLOS One
